# The changes of cardiac energy metabolism with sodium-glucose transporter 2 inhibitor therapy

**DOI:** 10.3389/fcvm.2023.1291450

**Published:** 2023-12-06

**Authors:** Sha Su, Xiang Ji, Tong Li, Yu Teng, Baofu Wang, Xiaowan Han, Mingjing Zhao

**Affiliations:** ^1^ Key Laboratory of Chinese Internal Medicine of Ministry of Education and Beijing, Dongzhimen Hospital Affiliated to Beijing University of Chinese Medicine, Beijing, China; ^2^ Department of Cardiology, Dongzhimen Hospital Affiliated to Beijing University of Chinese Medicine, Beijing, China

**Keywords:** sodium-glucose transporter 2 inhibitor, heart, energy metabolism, fatty acid, glucose, ketone body

## Abstract

**Background/aims:**

To investigate the specific effects of s odium-glucose transporter 2 inhibitor (SGLT2i) on cardiac energy metabolism.

**Methods:**

A systematic literature search was conducted in eight databases. The retrieved studies were screened according to the inclusion and exclusion criteria, and relevant information was extracted according to the purpose of the study. Two researchers independently screened the studies, extracted information, and assessed article quality.

**Results:**

The results of the 34 included studies (including 10 clinical and 24 animal studies) showed that SGLT2i inhibited cardiac glucose uptake and glycolysis, but promoted fatty acid (FA) metabolism in most disease states. SGLT2i upregulated ketone metabolism, improved the structure and functions of myocardial mitochondria, alleviated oxidative stress of cardiomyocytes in all literatures. SGLT2i increased cardiac glucose oxidation in diabetes mellitus (DM) and cardiac FA metabolism in heart failure (HF). However, the regulatory effects of SGLT2i on cardiac FA metabolism in DM and cardiac glucose oxidation in HF varied with disease types, stages, and intervention duration of SGLT2i.

**Conclusion:**

SGLT2i improved the efficiency of cardiac energy production by regulating FA, glucose and ketone metabolism, improving mitochondria structure and functions, and decreasing oxidative stress of cardiomyocytes under pathological conditions. Thus, SGLT2i is deemed to exert a benign regulatory effect on cardiac metabolic disorders in various diseases.

**Systematic review registration:**

https://www.crd.york.ac.uk/, PROSPERO (CRD42023484295).

## GRAPHICAL ABSTRACT

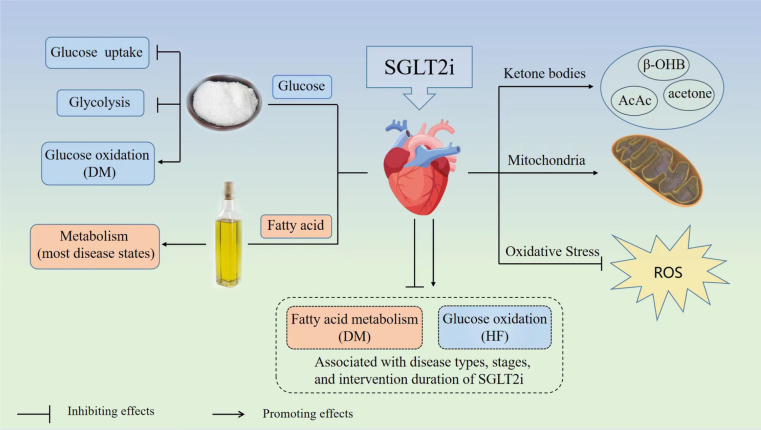
 Graphical abstracts of this systematic review. SGLT2i, sodium-glucose transporter 2 inhibitor; DM, diabetes mellitus; HF, heart failure; ROS, reactive oxygen species; β-OHB, β-hydroxybutyric acid; AcAc, acetoacetate.

## 1. Introduction

The heart maintains blood circulation of the whole body through uninterrupted systolic and diastolic functions; this process requires a large supply of ATP. Therefore, normal energy metabolism is fundamental for the physiological functions of the heart. Several diseases lead to abnormal energy metabolism, which is one of the major pathological mechanisms of the disrupted physiological functions of the heart. Therefore, regulating energy metabolism is crucial to improving cardiac physiological functions.

As an oral hypoglycemic medication, sodium-glucose transporter 2 inhibitor (SGLT2i) blocks glucose reabsorption in the renal proximal tubules, improving urine glucose excretion and regulating plasma glucose levels (1). Clinically, SGLT2i is mainly used for patients with type 2 diabetes mellitus (T2DM), chronic kidney disease (CKD) and heart failure (HF). Moreover, SGLT2i has been included in the treatment guidelines for heart failure with reduced ejection fraction (HFrEF) (2, 3) and can improve the cardiovascular prognosis of high-risk patients (4), such as reducing the risk of cardiovascular mortality, HF hospitalizations and adverse kidney events (5–7). The benefits are noticeable early after SGLT2i treatment, and the absolute risk is reduced markedly. Thus, SGLT2i may exert a salutary role in the treatment of HFrEF by reducing hyperemia through diuretic/natriuretic properties.

Notably, SGLT2i regulates energy substrates metabolism in the heart and blood circulation (8–13), and also has effects on oxidative stress, mitochondria structure and functions of the heart (14, 15). Although it has been widely used in various diseases due to its cardiac benefits, the specific effects of SGLT2i on cardiac energy metabolism have not been discussed thoroughly. Some studies had shown that SGLT2i promoted the metabolism of cardiac energy substrates such as fatty acid (FA) and glucose in pathological conditions, while it played an inhibitory role in the metabolism of cardiac energy substrates in other diseases, so the results were inconsistent and confusing. Therefore, a systematic review to explore the specific effects of SGLT2i on cardiac energy metabolism is essential.

Based on the existing literatures, we analyzed the specific effects of SGLT2i on cardiac FA, glucose, ketone metabolism, mitochondria structure and functions, oxidative stress in clinical patients and experimental animals with different diseases to explicate the value of clinical application of SGLT2i in cardiac energy metabolism.

## 2. Methods

The report of this systematic review was prepared based on the PRISMA 2020 Statements.

### 2.1. Search strategy

Eight electronic databases (PubMed, Web of Science, Embase, Cochrane, China National Knowledge Infrastructure, WanFang Data, VIP Database, and SinoMed) were searched from inception up to April 20, 2023. The search strategy used the following general terms as MeSH terms or free terms: “Sodium Glucose Transporter 2 Inhibitors”, “SGLT-2 Inhibitors”, “SGLT 2 Inhibitors”, “SGLT2 Inhibitors”, “Sodium-Glucose Transporter 2 Inhibitor”, “Sodium Glucose Transporter 2 Inhibitor”, “SGLT2 Inhibitor”, “Inhibitor, SGLT2”, “Gliflozins”, “Gliflozin”, “SGLT-2 Inhibitor”, “Inhibitor, SGLT-2”, “SGLT 2 Inhibitor”, “Empagliflozin”, “Dapagliflozin”, “Canagliflozin”, “Ertugliflozin”, “heart, Myocardium”, “Cardiac Muscle”, “Muscle, Heart”, “Heart Muscle”, “Heart Muscles”, “Muscles, Heart”, “Myocardia”, “Muscle, Cardiac”, “Cardiac Muscles”, “Muscles, Cardiac”, “Energy Metabolism”, “Energy Metabolisms”, “Metabolism, Energy”, “Metabolisms, Energy”, “Energy Expenditure”, “Energy Expenditures”, “Expenditure, Energy”, “Expenditures”, “Energy”, “Bioenergetics”, “Bioenergetic”. For this search, no restrictions or filters were used. The comprehensive search strategy was found in supplement materials. A thorough search of the review articles and references for each identified study was done in order to find further pertinent studies.

### 2.2. Inclusion and exclusion criteria

The inclusion criteria of the clinical studies were as follows: (1) Participants: Patients were treated with SGLT2i, with or without the control group; (2) Method of research: randomized controlled trials (RCTs), cohort studies and cross-sectional studies were included; (3) Indicators related to cardiac energy metabolism were detected.

The inclusion criteria of the animal studies were as follows: (1) Animals had to be treated with SGLT2i; (2) The research measures were pertinent to cardiac energy metabolism; (3) There was no restriction on language usage and the literatures should be published in peer-reviewed journals.

The exclusion criteria were as follows: (1) Articles with incomplete information; (2) Duplicate publications; (3) Reviews, meta-analysis, and corresponding/conference abstracts.

### 2.3. Quality assessment

Two authors evaluated the publications' quality separately, and the Cochrane Collaboration tool was utilized to assess the risk of bias (ROB) of RCTs. There are six types of bias in it: selection, performance, detection, attrition, reporting and other sources of bias. “High risk”, “low risk”, or “unclear risk” was used to describe the concentration of each project. The selection of research population, compatibility of the study groups, and measurement of exposure factors were the three criteria used by the Newcastle–Ottawa scale (NOS) to evaluate the quality of cohort studies. Each study received a score of 0–9. Cross-sectional studies were evaluated using the National Institutes of Health (NIH) Quality Assessment Tool (16, 17). The tool comprises 14 questions, and studies with scores <7 were classified as poor, 7–9 as fair, and >9 as good.

SYRCLE's ROB tool was applied to assess the quality of animal studies. A total of ten items from six projects were used as evaluation criteria: sequence generation (i.e., selection bias), baseline characteristics (i.e., selection bias), allocation concealment (i.e., selection bias), random housing (i.e., performance bias), blinding (i.e., performance bias), random outcome assessment (i.e., detection bias), blinding (i.e., detection bias), incomplete outcomes data (i.e., attrition bias), selective outcome reporting (i.e., reporting bias), and other sources of bias. Each item was categorized as either “unclear”, “low risk”, or “high risk” (18). After discussing with the corresponding author, the differences were settled through consensus.

### 2.4. Data extraction

Data were extracted by two authors independently from the included literatures employing a standardized sheet created specifically for this systematic review. The basic characteristics of clinical studies were collected, consisting of first author's name, year of publication, country, diseases types, study design, group and sample size, SGLT2i names and dose, intervention time of SGLT2i and types of energy metabolism indicators. As for animal studies, the basic information of identified studies was extracted, including first author's name, year of publication, country, sex/animal strain, models (methods), group, SGLT2i names and dose, starting time/intervention time of SGLT2i and types of energy metabolism indicators. Any disagreement was discussed and settled in a consensus meeting with the corresponding author.

### 2.5. Data analysis

Due to the high heterogeneity of the included literatures, we only compared the general trends of changes in FA, glucose, ketone metabolism, mitochondria structure and functions, and oxidative stress related to cardiac energy metabolism in both clinical and animal studies. The outcome measures, which compared the SGLT2i groups with the model groups, were recorded as a significant uptrend marker “↑” or a significant downtrend marker “↓”. Therefore, a qualitative synthesis was adopted for this systematic review.

## 3. Results

### 3.1. Search results and study selection

**Figure 1:**
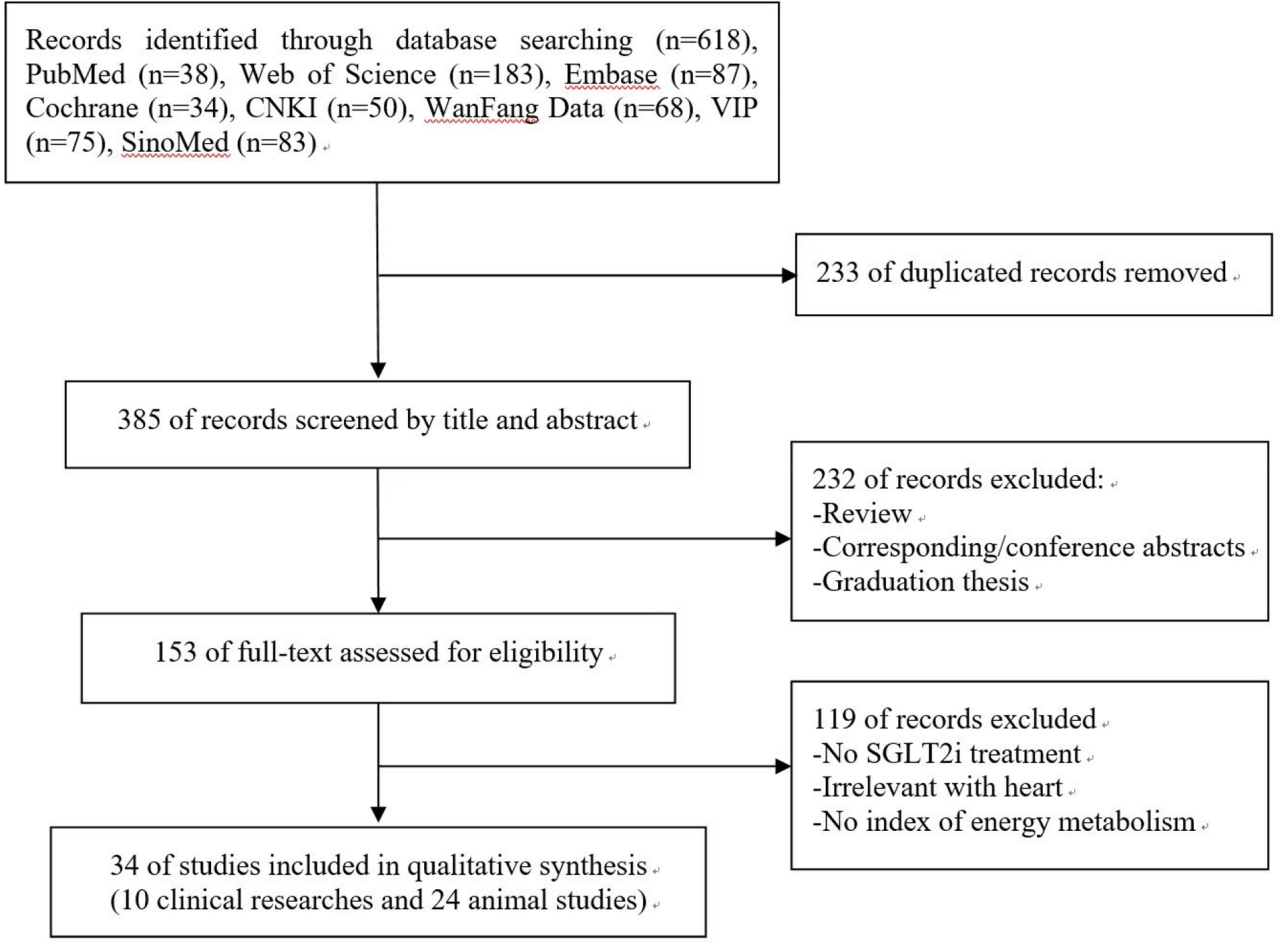
Flow chart of searching and screening studies.

A total of 618 records were identified from 8 electronic databases. After removing 233 duplicates, 385 potentially relevant literatures were assessed. Subsequently, 232 literatures were excluded after the evaluation of titles and abstracts. Of the 153 remaining literatures, we further excluded 119 after screening the full text. Finally, this systematic review contained 34 studies (10 clinical studies and 24 animal studies). The search procedure and study selection were depicted in the specific flow diagram (Figure 1).

### 3.2. Characteristics of included studies

#### 3.2.1. Characteristics of clinical studies

As shown in Table 1, the included studies comprised 5 RCTs, 3 cohort studies, and 2 cross-sectional studies. The diseases types included pre-diabetic insulin resistance, T2DM, HFrEF, heart failure with preserved ejection fraction (HFpEF), and aortic or mitral valve replacement surgery. The 10 studies' sample sizes varied from 13 to 1,299, with the majority of them containing both male and female participants. The age range was 52–72.1 years. Among the included clinical studies, Empagliflozin was used in 5 studies, Dapagliflozin in 3 and Canagliflozin in 2. SGLT2i was administrated orally in 9 studies but was used to process human cardiomyocytes in 1 study. The intervention duration of SGLT2i ranged from 2 to 52 weeks. The samples were mainly collected from plasma, and indicators related to FA, glucose, ketone metabolism, and oxidative stress were assessed.

**Table 1:** Characteristics of the included clinical studies.

Author, year, country	Diseases types	Study design	Group and sample size	SGLT2i name and dose	Intervention time of SGLT2i	Types of energy metabolism indicators
Veelen, 2023, the Netherlands (19)	Prediabetic insulin resistance	RCT (cross-over design)	14 (6–8 weeks washout)	Dapa (10 mg/day)	2 weeks	FA, GLU, KB
Hundertmark, 2023, England (20)	HFrEF and HFpEF	RCT	HFrEF: Placebo group: 19 SGLT2i group: 17 HFpEF: Placebo group: 18 SGLT2i group: 18	Empa (10 mg/day)	12 weeks	FA, KB, PCr: ATP
Berezin, 2023, Ukraine (21)	T2DM and HF	Cohort study	Male: 231 Female: 186	Dapa (10 mg/day)	6 months	GLU
Zannad, 2022, England (22)	HFrEF and HFpEF	Cross-sectional design	HFrEF: Placebo group: 299 SGLT2i group: 300 HFpEF: Placebo group: 268 SGLT2i group: 267	Empa	52 weeks	FA
Kondo, 2021, England (23)	Cardiac surgery	Cross-sectional design	51	Cana (10 μmol/L)	1 or 24 h	OS
Gaborit, 2021, France (24)	T2DM	RCT	Placebo group: 28 SGLT2i group: 28	Empa (10 mg/day)	12 weeks	FA, GLU, KB, PCr: ATP
Thirunavukarasu, 2021, England (25)	T2DM	Cohort study	Placebo group: 10 SGLT2i group: 18	Empa	12 weeks	PCr: ATP
Lauritsen, 2021, Denmark (11)	T2DM	RCT (cross-over design)	13 (1 w washout)	Empa (25 mg/day)	4 weeks	FA, GLU, KB
Oldgren, 2021, Sweden (26)	T2DM	RCT	Placebo group: 26 SGLT2i group: 27	Dapa (10 mg/day)	6 weeks	FA, GLU, KB
Polidori, 2017, Japan (27)	T2DM	cohort study	1,278	Cana (100 or 200 mg/day)	52 weeks	FA, GLU, KB

ATP, adenosine triphosphate; Cana, canagliflozin; Dapa, dapagliflozin; Empa, empagliflozin; FA, fatty acid; GLU, glucose; H, hour; HF, heart failure; HFpEF, heart failure with preserved ejection fraction; HFrEF, heart failure with reduced ejection fraction; KB, ketone body; OS, oxidative stress; PCr, phosphocreatine; RCT, randomized controlled trial; SGLT2i, sodium-glucose transporter 2 inhibitor; T2DM, type 2 diabetes mellitus.

#### 3.2.2. Characteristics of animal studies

A total of 24 animal studies were included in this systematic review. The detailed results were summarized in Table 2. The types of models included diabetes mellitus (DM) (4/24), myocardial infarction (MI) induced by left anterior descending coronary artery (LAD) ligation (2/24), cardiac pressure-overload by transverse aortic constriction (TAC) (4/24), ischemia-reperfusion (I/R) injury (6/24), gene knockout mouse (4/24), high-fat diet (HFD) (3/24), high-salt diet (1/24), high-carbohydrate diet (1/24), stress-induced cardiomyopathy (SCM) induced by isoproterenol (ISO) injected intraperitoneally (1/24), cardiotoxicity induced by doxorubicin (DOX) (1/24), and cardiac arrest (CA) induced by ventricular fibrillation (VF) (1/24), and 1 study (46) used normal animals without disease. The modeling time ranged from 10 min to 6 months. Among the 24 animal studies, 17 studies used Empagliflozin, 5 Dapagliflozin, 2 Canagliflozin, 2 Ertugliflozin, and 1 Sotagliflozin. The routes of SGLT2i administration included oral administration (*n* = 20), Langendorff heart infusion (*n* = 2), intraperitoneal injection (*n* = 1), and intravenous injection (*n* = 1). SGLT2i treatment duration ranged from 10 min to 12 weeks. The primary objective indicators in plasma samples were the same as in the included clinical studies, while animal studies included indicators related to energy metabolism in cardiac tissue.

**Table 2:** Characteristics of the included animal studies.

Author, year, country	Sex/animal strain	Models (methods)	Group	SGLT2i name and dose	Starting time and intervention time of SGLT2i	Types of energy metabolism indicators
Croteau, 2023, America (28)	Male mice	HFD	Sham, Model, Model + Ertu	Ertu	6 months/4 weeks	PCr: ATP
Chen, 2023, China (29)	Male rats	Cardiotoxicity (intraperitoneal injection of DOX)	Sham, Model, Model + Empa, Model + Meto	Empa (30 mg/kg/day)	4 weeks/4 weeks	Mito, OS, ATP
Xi, 2022, China (30)	Male rats	DCM (intraperitoneal injection of STZ)	Sham, Model, Model + Empa	Empa (30 mg/kg/day)	18 weeks/12 weeks	FA, Mito
Song, 2021, America (31)	Male mice	Adverse cardiac remodeling (LAD ligation), *Parkin* gene knockout	WT: Veh, Empa KO: Veh, Empa	Empa (10 mg/kg/day)	2 h/2 weeks	Mito
Shiraki, 2022, Japan (32)	Male mice	Dilated cardiomyopathy (heart and skeletal muscle-specific MnSOD-deficiency)	Sham, Model, Model + Empa	Empa (10 mg/kg/day)	NR/7 weeks	FA, GLU, KB, Mito
Nikolaou, 2022, Greece (33)	Male mice	LAD ligation /I/R	Model, Model + Empa, Model + Dapa, Model + Ertu	Empa (10 mg/kg/day), Dapa (9 mg/kg/day), Ertu (9.7 mg/kg/day)	2.5 h/1 week	GLU, Mito
Li, 2022, America (34)	Male mice	HF (TAC)	Sham, Sham + Empa, Model, Model + Empa	Empa (10 mg/kg/day)	2 weeks/4 weeks	GLU, Mito, OS
He, 2022, China (12)	Male rats	HFpEF (high-salt diet)	Sham, Model, Model + Cana	Cana (20 mg/kg/day)	12 weeks/12 weeks	FA, GLU, KB, Mito, OS, ATP
Cai, 2022, China (35)	Male mice	AKI (bilateral renal artery Ischemia I/R), *FUNDC1* gene knockout	WT: Sham, Model, Model + Empa KO: Sham, Model, Model + Empa	Empa (10 mg/kg/day)	72.5 h/1 week	Mito, ATP
Zhang, 2022, China (36)	Male rats	I/R (LAD ligation)	Sham, Model, Model + Empa, Model + Empa + EX527	Empa (2.5 mol/L)	2.7 h/10 min	OS
Shen, 2022, China (37)	Male mice	SCM (intraperitoneal injection of ISO)	Sham, Model, Dapa	Dapa (10 mg/kg/day)	NR/3 days	OS
Young, 2021, Australia (38)	Male mice	Cardiac pressure overload (HFD and TAC)	ND: Sham, Sham + Sota, Model, Model + Sota HFD: Sham, Sham + Sota, Model, Model + Sota	Sota (10 mg/kg/day)	HFD (1 week) + TAC(4 weeks)/7 weeks	GLU, KB
Trang, 2021, Taiwan, China (13)	Male rats	DM (intraperitoneal injection of STZ)	Sham, Model, Model + Empa, Model + Lira	Empa (10 mg/kg/day)	2 weeks/4 weeks	FA, GLU
Tan, 2021, China (39)	Male rats	CA (VF through a transoesophageal electrode)	Sham, Model, Model + Empa	Empa (10 mg/kg)	10 min/intraperitoneal injection	FA, GLU, KB, Mito, OS, ATP
Nikolaou, 2021, Greece (40)	Male mice	I/R (LAD)	Model, Model + Empa	Empa (10 mg/kg/day)	2.5 h/ Acute: 4 or 24 h Chronic: 6 weeks	GLU, OS
Li, 2021, America (41)	Male mice	Cardiac pressure overload (TAC)	Sham, Sham + Empa, Model, Model + Empa	Empa (10 mg/kg/day)	2 weeks/4 weeks	FA, GLU, KB, ATP
Gaborit, 2021, France (24)	Male mice	HFD	Sham, Model, Model + Empa	Empa (30 mg/kg/day)	4 weeks/12 weeks	FA, GLU, KB
Bai, 2021, China (42)	Female rats	I/R (LAD ligation) and T2DM (female Goto-Kakizaki rats)	Sham, Model, Model + Dapa	Dapa (1 mg/kg)	2.5 h/left femoral vein injection before I/R	FA, Mito, ATP
Li, 2020, China (43)	Male rats	Cardiac pressure overload (AC)	Sham, Sham + Dapa, Model, Model + Dapa	Dapa (10 mg/kg/day)	12 weeks/12 weeks	OS
Yurista, 2019, the Netherlands (44)	Male rats	MI (LAD ligation)	Sham + Veh, Sham + Empa, Model + Veh, Model + Empa-E, Model + Empa-L	Empa (30 mg/kg/day)	NR/ Early: 2 days Late: 2 weeks	FA, GLU, KB, Mito, OS, ATP
Santos-Gallego, 2019, America (15)	Female pigs	HF (balloon occlusion of LAD)	Model, Model + Empa	Empa (10 mg/kg/day)	2 h/8 weeks	FA, GLU, KB, ATP
Adingupu, 2019, Sweden (45)	Male mice	Early DM-insulin resistance (*leptin* gene knockout)	Sham, Model, Model + Empa	Empa (1.5 mg/kg/day)	NR/10 weeks	FA, GLU, KB
Uthman, 2018, the Netherlands (46)	Male mice	NR	Veh, Empa, Dapa, Cana	Empa (1 μmol/L), Dapa (1 μmol/L), Cana (3 μmol/L)	NR/30 min	ATP
Durak, 2018 Turkey (47)	Male rats	MetS (high-carbohydrate diet)	Sham, Model, Model + Dapa, Model + Insu	Dapag (5 mg/kg/day)	28 weeks/2 weeks	GLU, Mito, OS, ATP

AC, aortic coarctation; AKI, acute kidney injury; ATP, adenosine triphosphate; CA, cardiac arrest; Cana, canagliflozin; Dapa, dapagliflozin; DCM, diabetic cardiomyopathy; DM, diabetes mellitus; DOX, doxorubicin; Empa, empagliflozin; Ertu, ertugliflozin; EX527, SIRT1 inhibitor; FA, fatty acid; FUNDC1, FUN14 domain-containing protein 1; GLU, glucose; H, hour; HF, heart failure; HFD, high-fat diet; HFpEF, heart failure with preserved ejection fraction; I/R, ischemia-reperfusion; Insu, insulin; ISO, isoproterenol; KB, ketone body; KO, knockout; LAD, left anterior descending coronary artery; Lira, liraglutide; Meto, metoprolol; MetS, metabolic syndrome; MI, myocardial infarction; Min, minute; Mito, mitochondria; MnSOD, manganese superoxide dismutase; NR, not reported; OS, oxidative stress; PCr, phosphocreatine; RCT, randomized controlled trial; SCM, stress-induced cardiomyopathy; SGLT2i, sodium-glucose transporter 2 inhibitor; Sota, Sotagliflozin; STZ, streptozocin; TAC, transverse aortic constriction; T2DM, type 2 diabetes mellitus; Veh, vehicle; VF, ventricular fibrillation; WT, wild-type.

### 3.3. Quality assessment of included studies

A total of 5 RCTs were evaluated using the Cochrane evaluation tool, and the results were summarized in Supplementary Table S1 (11, 19, 20, 24, 26). The evaluation results showed that all of 5 studies used random allocation; and attrition bias, reporting bias and other sources of bias were low risks and detection bias were unclear. 4 studies had a low risk about performance bias (11, 19, 20, 26). Allocation concealment was implemented in 2 studies (20, 24). The methodological quality results of the 3 cohort studies evaluated by NOS were shown in Supplementary Table S2. The included studies' mean NOS score was 7 and the overall scores ranged from 66 to 89%. The 2 cross-sectional studies were assessed using the NIH Risk bias tool with scores of 8 (22) and 9 (23), respectively. The detailed results were shown in Supplementary Table S3. According to the assessment of SYRCLE's ROB tool, 24 animal studies reported that the selective outcome was low risk, while the sources of biases, such as animal breeder, investigator blinding, random outcome assessment, and other biases, were unclear. In most studies, the sources of risk regarding sequence generation (22/24), baseline characteristics (19/24), allocation concealment (20/24), and implementation of blinding of outcome assessors (22/24), were unclear. Most studies had a low risk of random housing and incomplete outcome data (14/24). The details of the ROB of the experimental studies were provided in Supplementary Table S4.

### 3.4. Outcome analysis

This systematic review was comprised of 34 studies in total. These consisted of 10 clinical studies, of which 7 studies were related to DM and 3 studies were related to HF. The 24 animal studies included 4 DM, 2 LAD ligation, 4 TAC, 6 I/R, 4 gene knockout models, and other models types. The main sources of samples collection included plasma and hearts of patients and animals. The energy metabolism-related indicators mainly included FA, glucose, ketone metabolism, mitochondria structure and functions, and oxidative stress.

#### 3.4.1. Effects of SGLT2i on cardiac FA metabolism

Of the 34 studies retrieved, 18 studies (7 clinical and 11 animal studies) addressed FA metabolism. The related indicators in plasma samples included the levels of free fatty acid (FFA), triglyceride (TG), total cholesterol (TC), high-density lipoprotein cholesterol (HDL-c), and low-density lipoprotein cholesterol (LDL-c). The FA metabolism-related indicators in heart samples were as follows: the content of FA translocase (CD36), carnitine palmitoyl transferase-1 (CPT-1), peroxisome proliferator-activated receptor γ coactivator-1α (PGC1-α), peroxisome proliferator-activated receptor α (PPARα), the ratio of phosphorylated-adenosine monophosphate-activated protein kinase to adenosine monophosphate-activated protein kinase (p-AMPK/AMPK), the ratio of phosphorylated-acetyl CoA carboxylase to acetyl CoA carboxylase (p-ACC/ACC), myocardial TG content, myocardial FA uptake, epicardial fat volume, the total number of lipid droplets in cardiomyocytes, and proteomics and metabolomics of myocardial tissue (details were shown in Table 3). Among these, 6 studies showed that SGLT2i increased the FA metabolism of the heart (12, 15, 30, 41, 42, 44), including the expressions of FA uptake-related transporter protein CD36, FA from cytoplasm into mitochondria related protein CPT-1, FA oxidation-related proteins (PGC1-α, PPARα, AMPK, and ACC); notably, the EF values of 5/6 studies (12, 30, 41, 42, 44) were >50% which were similar to the EF values of HFpEF. Cardiac FA metabolism was reduced in 1 study (13) and remained unchanged in 2 studies (11, 32). This phenomenon might be related to the types and stages of diseases. In addition, we could not determine the specific effect of SGLT2i on cardiac FA metabolism due to limited detection indicators in 9 studies (19, 20, 22, 24, 26, 27, 39, 45). Besides, 12 studies (4 clinical and 8 animal studies) documented body weight alterations. 4 studies (12, 24, 27, 45) reported weight loss. 7 studies (11, 15, 19, 20, 30, 32, 41) showed no differences in changes to body weight and 1 study (13) reported increases in body weight. Table 3 displayed the specific results.

**Table 3:** Effects of SGLT2i on cardiac FA metabolism in different diseases.

Author, year	Disease types/model (methods)	SGLT2i name and dose	Starting time/intervention time of SGLT2i	Changes in FA metabolism related indicators	Weight change
Xi, 2022 (30)	DCM (intraperitoneal injection of STZ)	Empa (30 mg/kg/day)	18 weeks/12 weeks	Plasma: TG↑, TC↑, HDL-c↑, LDL-c→ Heart: Proteomics↑[proteins associated with lipid metabolic processes such as lipid transport, lipoprotein metabolic process, lipid binding]	→
He, 2022 (12)	HFpEF (high-salt diet)	Cana (20 mg/kg/day	12 weeks/12 weeks	Plasma: TG→, TC→, LDL-c→ Heart: Metabolomics↑ [oxidative metabolic degradation products of FAs such as 3-HTA, 5-Zdodecenoic acid, nervonic acid, ricinoleic acid, ethyloleate, cholesterol sulfate, 3-hydroxycapric acid and DHA]	↓
Li, 2021 (41)	Cardiac pressure overload (TAC)	Empa (10 mg/kg/day)	2 weeks/4 weeks	Heart: FAO in whole hearts↑, the proteins expression of CD36↑, PPARα↑, p-AMPK/AMPK↑, p-ACC/ACC↑	→
Bai, 2021 (42)	I/R (LAD ligation) and T2DM (female Goto-Kakizaki rats)	Dapa (1 mg/kg)	2.5 h/left femoral vein injection before I/R	Heart: the mRNA expression of CPT-1↑, PGC1-α↑	NR
Yurista, 2019 (44)	MI (LAD ligation)	Empa (30 mg/kg/day)	NR/ Early: 2 days Late: 2 weeks	Heart: the mRNA expression of CPT1-α, PGC1-α↑	NR
Santos-allego, 2019 (15)	HF (balloon occlusion of LAD)	Empa (10 mg/kg/day)	2 h/8 weeks	Heart: FFA uptake↑, the proteins expression of CD36→, CPT1→, p-AMPK/AMPK↑, PGC-1α↑	→
Lauritsen, 2021 (11)	T2DM	Empa (25 mg/day)	NR/4 weeks	Plasma: FFA↑ Heart: the relative myocardial FFA uptake rate↓, FFA oxidation rate↓, FFA reesterification rate→, the absolute myocardial FFA uptake rate→, FFA oxidation rate→, FFA reesterification rate→	→
Trang, 2021 (13)	DM (intraperitoneal injection of STZ)	Empa (10 mg/kg/day)	2 weeks/4 weeks	Plasma: TG↓, TC→, HDL-c→, LDL-c↓, FFAs↓ Heart: the proteins expression of CD36↓, CPT-1β↓, pACC↓, PGC-1α↓, p-AMPKα2/AMPKα2↑	↑
Shiraki, 2022 (32)	Dilated cardiomyopathy (heart and skeletal muscle-specific MnSOD-deficiency)	Empa (10 mg/kg/day)	NR/7 weeks	Heart: the fat tracer 125I-BMIPP→, the proteins expression of CD36→	→
Veelen, 2023 (19)	Prediabetic insulin resistant	Dapa (10 mg/day)	NR/2 weeks	Plasma: FFA→, AUC for 24-hour free fatty acid profiles, as well as day- and night-time profiles↑, fasting free glycerol levels→, AUC for 24-hour free glycerol profiles, as well as day- and night-time profiles→	→
Hundert-mark, 2023 (20)	HFrEF and HFpEF	Empa (10 mg/day)	NR/12 weeks	Plasma: FFA→ Heart: myocardial TG↓	→
Zannad, 2022 (22)	HFrEF and HFpEF	Empa	NR/52 weeks	Plasma: Proteomics↑[AFABP4]	NR
Gaborit, 2021 (24)	T2DM	Empa (10 mg/day)	NR/12 weeks	Heart: epicardial fat volume→, myocardial TG→	↓
Oldgren, 2021 (26)	T2DM	Dapa (10 mg/day)	NR/6 weeks	Plasma: FFA→ Heart: FA uptake→	NR
Polidori, 2017 (27)	T2DM	Cana (100 or 200 mg/day)	NR/52 weeks	Plasma: TG↓, FFA↑	↓
Tan, 2021 (39)	CA (VF through a transoesophageal electrode)	Empa (10 mg/kg)	10 min/intraperitoneal injection	Heart: the total number of lipid droplets↓	NR
Gaborit, 2021 (24)	HFD	Empa (30 mg/kg/day)	4 weeks/12 weeks	Heart: myocardial fat content→, myocardial TG→	↓
Adingupu, 2019 (45)	Early DM-insulin resistant (*leptin* gene knockout)	Empa (1.5 mg/kg/day)	NR/10 weeks	Plasma: TG→, TC↓	↓

ACC, acetyl CoA carboxylase; AFABP4, adipocyte fatty acid-binding protein 4; AMPK, adenosine monophosphate-activated protein kinase; AUC, area under the curve; BMIPP: β-methyl-P-iodophenyl-penta-decanoic acid; CA, cardiac arrest; Cana, canagliflozin; CD36, fatty acid translocase; CPT-1, carnitine palmitoyl transferase-1; Dapa, dapagliflozin; DCM, diabetic cardiomyopathy; DHA, docosahexaenoic acid; DM, diabetes mellitus; Empa, empagliflozin; FA, fatty acid; FAO, fatty acid oxidation; FFA, free fatty acid; H, hour; HDL-c, high-density lipoprotein cholesterol; HF, heart failure; HFD, high-fat diet; HFpEF, heart failure with preserved ejection fraction; HFrEF, heart failure with reduced ejection fraction; I/R, ischemia-reperfusion; LAD, left anterior descending coronary artery; LDL-c, low-density lipoprotein cholesterol; MI, myocardial infarction; Min, minute; MnSOD, manganese superoxide dismutase; NR, not reported; PGC1-α, peroxisome proliferator-activated receptor γ coactivator-1α; PPARα, peroxisome proliferator-activated receptor α; p-ACC, phosphor-acetyl CoA carboxylase; p-AMPK, phosphor-adenosine monophosphate-activated protein kinase; SGLT2i, sodium-glucose transporter 2 inhibitor; STZ, streptozocin; TAC, transverse aortic constriction; TC, total cholesterol; TG, triglyceride; T2DM: type 2 diabetes mellitus; VF, ventricular fibrillation; 3-HTA,3-hydroxy-tetradecanoic acid.

#### 3.4.2. Effects of SGLT2i on cardiac glucose metabolism

As shown in Table 4, 20 studies, including 6 clinical and 14 animal studies, were related to glucose metabolism. The indexes evaluated in plasma samples involved blood glucose and lactic acid (LA) levels. The indicators in myocardium tissue consisted of glucose uptake, glucose oxidation and glycolysis, specifically including the content of glucose transporter 4 (GLUT4) protein, pyruvate dehydrogenase (PDH), pyruvate dehydrogenase kinase 4 (PDK4), and lactate dehydrogenase (LDH). The results showed that myocardial glucose uptake ability decreased in 3 studies (11, 15, 41), while 2 studies (13, 44) demonstrated a significant increase in GLUT4 protein expression in the Empagliflozin group, regardless of T2DM. A total of 3 studies (13, 41, 44) reported that Empagliflozin upregulated glucose oxidation, while some studies (12, 15) put forth an opposite conclusion. Several animal studies (32, 39, 41) reported a decreased serum LA level. Another animal study suggested that Empagliflozin normalized myocardial LA consumption compared to higher net myocardial LA production in the control group (15). However, the level of serum LA remained unchanged in 2 clinical studies (11, 26). In addition, 12 studies could not decide the effect of SGLT2i on cardiac glucose metabolism due to incomplete indicators (19, 21, 24, 26, 27, 33, 34, 38, 40, 45, 47).

**Table 4:** Effects of SGLT2i on cardiac glucose metabolism in different diseases.

Author, year	Disease types/model (methods)	SGLT2i name and dose	Starting time/intervention time of SGLT2i	Changes in glucose metabolism related indicators
Trang, 2021 (13)	DM (intraperitoneal injection of STZ)	Empa (10 mg/kg/day)	2 weeks/4 weeks	Plasma: BG↓ Heart: GLUT4↑, pIRS1/IRS1↑, pAkt/Akt↑
Yurista, 2019 (44)	MI (LAD)	Empa (30 mg/kg/day)	NR/ Early: 2 days Late: 2 weeks	Heart: the mRNA expression of GLUT4↑ and PDK4↓, PDH↑
Lauritsen, 2021 (11)	T2DM	Empa (25 mg/day)	NR/4 weeks	Plasma: BG↓, LA↓ Heart: the relative myocardial glucose uptake rate↓, the absolute myocardial glucose uptake rate↓
Li, 2021 (41)	Cardiac pressure overload (TAC)	Empa (10 mg/kg/day)	2 weeks/4 weeks	Plasma: BG→, LA↓ Heart: glucose oxidation↑, glycolysis↓, glucose uptake↓
Santos-Gallego, 2019 (15)	HF (balloon occlusion of LAD)	Empa (10 mg/kg/day)	2 h/8 weeks	Plasma: BG→ Heart: glucose uptake↓, LA↓, LDH↓, PDH↓
He, 2022 (12)	HFpEF (high-salt diet)	Cana (20 mg/kg/day)	12 weeks/12 weeks	Plasma: BG→ Heart: PDK4↑, D-glucose↓, glyceraldehyde↓, D-xylitol↓
Shiraki, 2022 (32)	Dilated cardiomyopathy (heart and skeletal muscle-specific MnSOD-deficiency)	Empa (10 mg/kg/day)	NR/7 weeks	Plasma: CHO↑, LA↓; the glucose tracer 3H-2DG→ Heart: GLUT4→
Tan, 2021 (39)	CA (VF through a transoesophageal electrode)	Empa (10 mg/kg)	10 min/intraperitoneal injection	Plasma: BG↓, LA↓
Oldgren, 2021 (26)	T2DM	Dapa (10 mg/day)	NR/6 weeks	Heart: HbA1c↓, BG↓, LA→
Veelen, 2023 (19)	Prediabetic insulin resistance	Dapa (10 mg/day)	NR/2 weeks	Plasma: BG→, CHO↓, AUC for 24-h plasma glucose profiles↓
Berezin, 2023 (21)	T2DM and HF	Empa (10 mg/day)	NR/6 months	Plasma: BG→, HbAc1→
Gaborit, 2021 (24)	T2DM	Empa (10 mg/day)	NR/12 weeks	Plasma: BG↓, HbAc1↓
Polidori, 2017 (27)	T2DM	Cana (100 or 200 mg/day)	NR/52 weeks	Plasma: BG↓, HbAc1↓
Nikolaou, 2022 (33)	LAD ligation /I/R	Empa (10 mg/kg/day), Dapa (9 mg/kg/day), Ertu (9.7 mg/kg/day)	2.5 h/1 week	Plasma: BG→
Li, 2021 (41)	Cardiac pressure overload (TAC)	Empa (10 mg/kg/day)	2 weeks/4 weeks	Plasma: BG→
Young, 2021 (38)	Cardiac pressure overload (HFD and TAC)	Sota (10 mg/kg/day)	HFD (1 week)+TAC (4 weeks)/7 weeks	Plasma: BG↓
Nikolaou, 2021 (40)	I/R (LAD)	Empa (10 mg/kg/day)	2.5 h/ Acute: 4 or 24 h Chronic: 6 weeks	Plasma: BG→
Gaborit, 2021 (24)	HFD	Empa (30 mg/kg/day)	4 weeks/12 weeks	Plasma: BG↓, HbAc1↓
Adingupu, 2019 (45)	Early DM-insulin resistance (*leptin* gene knockout)	Empa (1.5 mg/kg/day)	NR/10 weeks	Plasma: HbAc1↓
Durak, 2018 (47)	MetS (high-carbohydrate diet)	Empa (30 mg/kg/day)	NR/ Early: 2 days Late: 2 weeks	Plasma: BG↓

Akt, protein kinase B; AUC, area under the curve; BG, blood glucose; CA, cardiac arrest; Cana, canagliflozin; CHO, carbohydrate; Dapa, dapagliflozin; DM, diabetes mellitus; Empa, empagliflozin; Ertu, ertugliflozin; GLUT4, glucose transporter 4; H, hour; HF, heart failure; HFD, high-fat diet; HFpEF, heart failure with preserved ejection fraction; IRS1, insulin receptor substrate 1; I/R, ischemia-reperfusion; LA, lactate; LAD, left anterior descending coronary artery; LDH, lactate dehydrogenase; MetS, metabolic syndrome; MI, myocardial infarction; Min, minute; MnSOD, manganese superoxide dismutase; NR, not reported; pAkt, phosphorylated protein kinase B; PDH, pyruvate dehydrogenase; PDK4, pyruvate dehydrogenase kinase 4; pIRS1, phosphorylated insulin receptor substrate 1; SGLT2i, sodium-glucose transporter 2 inhibitor; Sota, sotagliflozin; TAC, transverse aortic constriction; T2DM, type 2 diabetes mellitus; VF, ventricular fibrillation.

#### 3.4.3. Effects of SGLT2i on cardiac ketone metabolism

Of the 34 studies included in this systematic review, 15 studies (6 clinical and 9 animal studies) addressed ketone metabolism. The related indexes of ketone metabolism in plasma samples included the levels of ketone body (KB), total ketone bodies (TKB), β-hydroxybutyrate (β-OHB), and acetoacetate (AcAc). The ketone metabolic indicators in heart samples included the content of KB, TKB, β-OHB, KB transporter monocarboxylate transporter 1 (MCT1), monocarboxylate transporter 2 (MCT2), β-hydroxybutyrate dehydrogenase 1 (BDH1), β-hydroxybutyrate dehydrogenase 2 (BDH2), and succinyl-CoA: 3-ketoacid CoA transferase (SCOT). As illustrated in Table 5, 10 studies showed that SGLT2i increased the levels of ketone bodies (KBs) in plasma (11, 12, 15, 19, 24, 27, 39, 44, 45); 4 studies showed that SGLT2i increased the cardiac ketone metabolism (12, 15, 39, 44); 2 studies did not show any significant change, which might be related to the types of disease models; 4 studies showed no change in plasma ketone levels after SGLT2i treatment (20, 26, 32, 41). The detailed results were shown in Table 5.

**Table 5:** Effects of SGLT2i on cardiac ketone metabolism in different diseases.

Author, year	Disease types/model (methods)	SGLT2i name and dose	Starting time/intervention time of SGLT2i	Changes in KB metabolism related indicators
He, 2022 (12)	HFpEF (high-salt diet)	Cana (20 mg/kg/day)	12 weeks/12 weeks	Plasma: β-OHB↑ Heart: β-OHB↑, the proteins expression of ketogenic protein Hmgcs2↑ and BDH1↓
Tan, 2021 (39)	CA (VF through a transoesophageal electrode)	Empa (10 mg/kg)	10 min/intraperitoneal injection	Plasma: β-OHB↑ Heart: β-OHB↑, the protein expression of BDH1↑
Yurista, 2019 (44)	MI (LAD ligation)	Empa (10 mg/kg/day)	NR/ Early: 2 days Late: 2 weeks	Plasma: KB↑, urinary ketone excretion↑ Heart: the mRNA expression of MCT1↑ and BDH1↑, the protein expression of SCOT↑
Santos-Gallego, 2019 (15)	HF (balloon occlusion of LAD)	Empa (10 mg/kg/day)	2 h/8 weeks	Plasma: KB↑, KB plasma/myocardium ratio↑ Heart: KB uptake↑, the activity and expression of SCOT↑, the protein expression of BDH1↑
Veelen, 2023 (19)	Prediabetic insulin resistance	Dapa (10 mg/day)	NR/2 weeks	Plasma: AUC for β-OHB↑
Gaborit, 2021 (24)	T2DM	Empa (10 mg/day)	NR/12 weeks	Plasma: KB↑
Lauritsen, 2021 (11)	T2DM	Empa (25 mg/day)	NR/4 weeks	Plasma: β-OHB↑
Polidori, 2017 (27)	T2DM	Cana (100 or 200 mg/day)	NR/52 weeks	Plasma: β-OHB↑, AcAc↑,TKB↑
Gaborit, 2021 (24)	HFD	Empa (30 mg/kg/day)	4 weeks/12 weeks	Plasma: KB↑, β-OHB↑ Heart: the mRNA expression of bdh1→, bdh2→, hmgcs2→ and oxct1→
Adingupu, 2019 (45)	Early DM-insulin resistance (*leptin* gene knockout)	Empa (1.5 mg/kg/day)	NR/10 weeks	Plasma: β-OHB↑
Young, 2021 (38)	Cardiac pressure overload (HFD and TAC)	Sotag (10 mg/kg/day)	HFD(1 weeks) +TAC(4 weeks)/7 weeks	Plasma: β-OHB→ Heart: the protein expression of Mct2→ and BDH1→
Hundertmark, 2023 (20)	HFrEF and HFpEF	Empa (10 mg/day)	NR/12 weeks	Plasma: β-OHB→
Oldgren, 2021 (26)	T2DM	Dapa (10 mg/day)	NR/6 weeks	Plasma: β-OHB→
Shiraki, 2022 (32)	Dilated cardiomyopathy (heart and skeletal muscle-specific MnSOD-deficiency)	Empa (10 mg/kg/day)	NR/7 weeks	Plasma: β-OHB→
Li, 2021 (41)	Cardiac pressure overload (TAC)	Empa (10 mg/kg/day)	2 weeks/4 weeks	Plasma: KB→

AcAc, acetoacetate; AUC, area under the curve; BDH1, β-hydroxybutyrate dehydrogenase 1; Bdh2, β-hydroxybutyrate dehydrogenase 2; β-OHB, β-hydroxybutyric acid; CA, cardiac arrest; Cana, canagliflozin; Dapa, dapagliflozin; DM, diabetes mellitus; Empa, empagliflozin; H, hour; HF, heart failure; HFD, high-fat diet; HFpEF, heart failure with preserved ejection fraction; HFrEF, heart failure with reduced ejection fraction; hmgcs2 and oxct1, the enzymes involved in the KB metabolism; KB, ketone body; LAD, left anterior descending coronary artery; MCT1, monocarboxylate transporter 1; MI, myocardial infarction; Min, minute; MnSOD, manganese superoxide dismutase; NR, not reported; SCOT, succinyl-CoA:3-ketoacid CoA transferase; SGLT2i, sodium-glucose transporter 2 inhibitor; Sota, sotagliflozin; TAC, transverse aortic constriction; TKB, total ketone bodies; T2DM: type 2 diabetes mellitus; VF, ventricular fibrillation.

#### 3.4.4. Effects of SGLT2i on mitochondria structure and functions of the heart

This systematic review consisted of 12 animal studies related to cardiac mitochondria structure and functions. The main detection indicators included mitochondria number, morphology, the average area, mitochondria membrane potential (MMP), the baseline oxygen consumption rate (OCR), the activity and oxidative phosphorylation (OXPHOS) capacity of mitochondria respiratory complexes I–III (COX I–III), the mitochondria DNA (mtDNA), the content of pro-fission dynein-related protein 1 (DRP1), mitochondria fission 1 protein (FIS1), mitochondria pro-fusion proteins mitofusin 1 (MFN-1), mitofusin 2 (MFN-2), optic nerve atrophy 1 (OPA1), p-AMPK, sirtuin-1 (SIRT1), and PGC-1α. These studies confirmed the advantages of SGLT2i treatment on cardiac mitochondria structure and functions. Table 6 provided a full description of the outcomes.

**Table 6:** Effects of SGLT2i on mitochondria structure and functions of the heart in different diseases.

Author, year	Disease types/model (methods)	SGLT2i name and dose	Starting time/intervention time of SGLT2i	Changes in cardiomyocytes mitochondria structure and functions related indicators
Chen, 2023 (29)	Cardiotoxicity (intraperitoneal injection of DOX)	Empa (30 mg/kg/day)	4 weeks/4 weeks	The proteins expression of p-AMPK↑, p-AMPK/AMPK ratio↑, SIRT-1↑, PGC-1α↑
Xi, 2022 (30)	DCM (intraperitoneal injection of STZ)	Empa (30 mg/kg/day)	18 weeks/12 weeks	Pleomorphic mitochondria, lipid deposition
Song, 2022 (31)	Adverse cardiac remodeling (LAD ligation), *Parkin* gene knockout	Empa (10 mg/kg/day)	2 h/2 weeks	Baseline mitochondrial oxygen consumption and maximal respiratory capacity↑, mitochondrial proteins↑[the 5 OXPHOS complexes, as well as COX IV, TOM20 and Mfn2]
Shiraki, 2022 (32)	Dilated cardiomyopathy (Heart and skeletal muscle-specific MnSOD-deficiency)	Empa (10 mg/kg/day)	NR/7 weeks	Complex I + II-linked OXPHOS capacity↑, complex II-linked OXPHOS capacity↑, the proteins expression of COX IV↑ and VDAC-1↑, the mtDNA content of the myocardium↑
Nikolaou, 2022 (33)	LAD ligation /I/R	Empa (10 mg/kg/day), Dapa (9 mg/kg/day), Ertu (9.7 mg/kg/day)	2.5 h/1 week	Complex I + II-linked OXPHOS capacity↑
Li, 2022 (34)	HF (TAC)	Empa (10 mg/kg/day)	2 weeks/4 weeks	Mitochondrial number and mean area↑, the proteins and mRNA expression of PGC1α↑, NRF-1↑, TFAM↑ and COX1↑
He, 2022 (12)	HFpEF (high-salt diet)	Cana (20 mg/kg/day)	12 weeks/12 weeks	The proteins expression of p-AMPK↑, SIRT1↑ and PGC-1α↑, the mRNA expression of SIRT1↑ and PGC-1α↑
Cai, 2022 (35)	AKI (bilateral renal arteryIschemia I/R), *FUNDC1* gene knockout	Empa (10 mg/kg/day)	72.5 h/1 week	The mitochondrial length↑, the proportion of rounded mitochondria↓, mitochondria swelled irregularly↓, cristae fractured and fuzzy↓, the duration of mPTP opening↓, MMP↑, the mtDNA copy number and transcription↑, the baseline OCR↑, the activities of mitochondrial respiratory complexes I-III↑, the mRNA expression of Drp1↓, Fis1↓, Mfn2↑ and Opa1↑
Tan, 2021 (39)	CA (VF through a transoesophageal electrode)	Empa (10 mg/kg)	10 min/intraperitoneal injection	Mitochondrial area↓, the protein expression of Drp1↓, the number of IMJs↑, the activity of mitochondrial respiratory complexes I↑, mitochondrial RCR↑
Bai, 2021 (42)	I/R (LAD ligation) and T2DM (female Goto-Kakizaki rats)	Dapa (1 mg/kg)	2.5 h/left femoral vein injection before I/R	MMP↑, the proteins expression of MFN2↑, OPA1↑, DRP1↓
Yurista, 2019 (44)	MI (LAD ligation)	Empa (30 mg/kg/day)	NR/ Early: 2 days Late: 2 weeks	MtDNA damage↓, mtDNA/nDNA ratio↑, the protein expression of PGC1-α↑
Durak, 2018 (47)	MetS (high-carbohydrate diet)	Dapa (5 mg/kg/day)	28 weeks/2 weeks	MMP↑, the proteins expression of Mfn-1↓, Mfn-2↑, Fish-1↓, Mfn-1/Mfn-2 ratio↓

AKI, acute kidney injury; AMPK, adenosine monophosphate-activated protein kinase; CA, cardiac arrest; Cana, canagliflozin; COX IV and TOM20, the OXPHOS complexes; COX1, cyclooxygenase1; Dapa, dapagliflozin; DCM, diabetic cardiomyopathy; DOX, doxorubicin; Drp1, dynein-related protein 1; Empa, empagliflozin; Ertu, ertugliflozin; Fis1, mitochondrial fission 1 protein; FUNDC1, FUN14 domain-containing protein 1; H, hour; HF, heart failure; HFpEF, heart failure with preserved ejection fraction; IMJs, intermitochondrial junctions; I/R, ischemia-reperfusion; LAD, left anterior descending coronary artery; MetS, metabolic syndrome; Mfn, mitofusin; MI, myocardial infarction; Min, minute; MMP, mitochondrial membrane potential; MnSOD, manganese superoxide dismutase; mPTP, mitochondrial permeability transition pore; mtDNA, mitochondrial DNA; nDNA, nuclear DNA; NR, not reported; NRF-1, nuclear respiratory factor 1; OCR, oxygen consumption rate; Opa1, optic nerve atrophy 1; OXPHOS, oxidative phosphorylation; p-AMPK, phosphor-adenosine monophosphate-activated protein kinase; PGC1-α, peroxisome proliferator-activated receptor γ coactivator-1α; RCR, respiratory control ratio; SGLT2i, sodium-glucose transporter 2 inhibitor; SIRT-1, sirtuin-1; STZ, streptozocin; TAC, transverse aortic constriction; TFAM, transcription factor A; T2DM: type 2 diabetes mellitus; VDAC-1, the mitochondrial outer membrane ion channel; VF, ventricular fibrillation.

#### 3.4.5. Effects of SGLT2i on oxidative stress of the heart

As shown in Table 7, 11 studies (1 clinical study and 10 animal studies) were connected with cardiac oxidative stress. The indicators included reactive oxygen species (ROS), superoxide dismutase (SOD), malondialdehyde acid (MDA), NADPH oxidase (NOX), and advanced oxidation protein product (AOPP). The disease types included HF, I/R, SCM, metabolic syndrome (MetS), and so on. 1 study showed that Canagliflozin improved oxidative stress in myocardial tissue (23). Reportedly, the treatment with Empagliflozin or Dapagliflozin greatly attenuated MDA and elevated SOD levels in myocardial tissue (29, 36, 37, 40, 43). 4 studies (34 36, 39, 47), revealed markedly inhibited cardiac ROS production. The expression of AOPP and NOX4 proteins was reduced in 2 studies (12, 44).

**Table 7:** Effects of SGLT2i on oxidative stress of the heart in different diseases.

Author, year	Disease types/model (methods)	SGLT2i name and dose	Starting time/intervention time of SGLT2i	Changes in oxidative stress related indicators
Kondo, 2021 (23)	Cardiac surgery (aortic or mitral-valve replacement)	Cana (10 μmol/L)	NR/1 or 24 h	Rac1↓, NOX2↓, BH4↑, BH2↓, pAMPKα2↑, pACC↑, pERK→, pAKT→
Chen, 2023 (29)	Cardiotoxicity (intraperitoneal injection of DOX)	Empa (30 mg/kg/day)	4 weeks/4 weeks	MDA↓, SOD↑, CAT↑
Li, 2022 (34)	HF (TAC)	Empa (10 mg/kg/day)	2 weeks/4 weeks	H_2_O_2_↓, cardiomyocyte superoxide↓, the protein expression of HO-1↑, NRF-2↑, the mRNA expression of NRF-2→, HO-1→, Catalase→, GCLM→
He, 2022 (12)	HFpEF (high-salt diet)	Cana (20 mg/kg/day)	12 weeks/12 weeks	AOPP↓, NOX4↓
Zhang, 2022 (36)	I/R (LAD)	Empa (2.5 mol/L)	2.7 h/10 min	SIRT1↑, gp91↓, ROS↓, MDA↓, SOD↑, SDH↑
Shen, 2022 (37)	SCM (intraperitoneal injection of ISO)	Dapa (10 mg/kg/day)	NR/3 days	MDA↓, GSH-Px↓, SOD↑
Tan, 2021 (39)	CA (VF through a transoesophageal electrode)	Empa (10 mg/kg)	10 min/intraperitoneal injection	4-HNE↓, 8-OHdG positive cells↓, ROS↓
Nikolaou, 2021 (40)	I/R (LAD)	Empa (10 mg/kg/day)	2.5 h/ Acute: 4 or 24 h Chronic: 6 weeks	MDA↓, PCs↓, SOD2↑, NOS2↑, NOX2 mRNA↑, p(Y705) STAT-3/t STAT-3 dipolymer↑
Li, 2020 (43)	Cardiac pressure overload (AC)	Dapa (10 mg/kg/day)	12 weeks/12 weeks	MDA↓, SOD↑
Yurista, 2019 (44)	MI (LAD)	Empa (30 mg/kg/day)	NR/ Early: 2 days Late: 2 weeks	AOPP↓, NOX2↓
Durak, 2018 (47)	MetS (high-carbohydrate diet)	Dapa (5 mg/kg/day)	28 weeks/2 weeks	ROS↓, RNS↓, SH→, oxidized SH↓

AC, aortic coarctation; AOPP, advanced oxidation protein product; BH, tetrahydrobiopterin; CA, cardiac arrest; Cana, canagliflozin; CAT, catalase; Dapa, dapagliflozin; DOX, doxorubicin; Empa, empagliflozin; GCLM, glutamate-cysteine ligase modifier subunit; GSH-Px, glutathione peroxidase; H, hour; HF, heart failure; HFpEF, heart failure with preserved ejection fraction; HO-1, heme oxygenase-1; ISO, Isoproterenol; I/R, ischemia-reperfusion; LAD, left anterior descending coronary artery; MDA, malondialdehyde acid; MetS, metabolic syndrome; MI, myocardial infarction; Min, minute; NOS, nitric oxide synthase; NOX, NADPH oxidases; NR, not reported; NRF-2, nuclear factor erythroid 2–related factor 2; pACC, phosphorylated acetyl coA carboxylase; pAKT, phosphorylated protein kinase B; pAMPKα2, phosphorylated adenosine monophosphate-activated protein kinase α2; pERK, phosphorylated extracellular regulated protein kinases; PCs, protein carbonyls; Rac1, ras-related C3 botulinum toxin substrate 1; RNS, reactive nitrogen species; ROS, reactive oxygen species; SCM, stress-induced cardiomyopathy; SDH, succinate dehydrogenase; SGLT2i, sodium-glucose transporter 2 inhibitor; SIRT, Sirtuins; SOD, superoxide dismutase; STAT, signal transducer and activator of transcription; SH, protein thiol; TAC, transverse aortic constriction; VF, ventricular fibrillation; 4-HNE, 4hydroxynonenal; 8-OHdG, an index of oxidative DNA damage.

#### 3.4.6. Effects of SGLT2i on cardiac energy metabolism in DM and HF

This systematic review covered a total of 12 studies pertaining to HF (3 clinical and 9 animal studies) and 11 studies pertaining to DM (7 clinical and 4 animal studies). Subsequently, we summarized the indicators related to energy metabolism as class indicators, including: FA metabolism (lipid levels in plasma and the content of proteins related to FA metabolism in the heart), glucose metabolism (cardiac glucose uptake, glucose oxidation, and glycolysis), KB metabolism (the level of KB in the circulation and the heart), myocardial mitochondria structure and functions, and oxidative stress in cardiomyocytes. The outcomes demonstrated that SGLT2i decreased the absorption of glucose and glycolysis, increased the circulating and cardiac level of KB in DM and HF, improved mitochondria structure and functions, and reduced oxidative stress in cardiomyocytes. In addition, SGLT2i increased the cardiac glucose oxidation in DM and FA metabolism in HF. However, SGLT2i had inconsistent regulatory effects on FA metabolism in DM and glucose oxidation in HF. Finally, because of the differences in the research purpose of each study, indicators related to energy metabolism were not mentioned in some literatures. The detailed results were shown in Tables 8, 9.

**Table 8:** Effects of SGLT2i on cardiac energy metabolism in DM.

Author, year	Disease types/model (methods)	SGLT2i name and dose	Starting time/intervention time of SGLT2i	FA	GLU	KB	Mito	OS
Veelen, 2023 (19)	Prediabetic insulin resistance	Dapa (10 mg/day)	NR/2 weeks	NR	NR	Plasma↑	NR	NR
Berezin, 2023 (21)	T2DM and HF	Dapa (10 mg/day)	NR/6 months	NR	NR	NR	NR	NR
Gaborit, 2021 (24)	T2DM	Empa (10 mg/day)	NR/12 weeks	NR	NR	Plasma↑	NR	NR
Thirunavukarasu, 2021 (25)	T2DM	Empa	NR/12 weeks	NR	NR	NR	NR	NR
Lauritsen, 2021 (11)	T2DM	Empa (25 mg/day)	NR/4 weeks	NR	Glucose uptake↓, glycolysis↓	Plasma↑	NR	NR
Oldgren, 2021 (26)	T2DM	Dapa (10 mg/day)	NR/6 weeks	NR	Glycolysis→	Plasma↑	NR	NR
Polidori, 2017 (27)	T2DM	Cana (100 or 200 mg/day)	NR/52 weeks	NR	NR	Plasma↑	NR	NR
Xi, 2022 (30)	DCM (intraperitoneal injection of STZ)	Empa (30 mg/kg/day)	18 weeks/12 weeks	Plasma↑, heart↑	NR	NR	Better	NR
Trang, 2021 (13)	DM (intraperitoneal injection of STZ)	Empa (10 mg/kg/day)	2 weeks/4 weeks	Plasma↓, heart↓	Glucose oxidation↑	NR	NR	NR
Bai, 2021 (42)	I/R(LAD) and T2DM (female Goto-Kakizaki rats)	Dapa (1 mg/kg)	2.5 h/left femoral vein injection before I/R	Heart↑	NR	NR	Better	NR
Uthman, 2018 (46)	NR	Empa (1 μmol/L), Dapa (1 μmol/L), Cana (3 μmol/L)	NR/30 min	NR	NR	Plasma↑	NR	NR

Cana, canagliflozin; Dapa, dapagliflozin; DCM, diabetic cardiomyopathy; DM, diabetes mellitus; Empa, empagliflozin; FA, fatty acid metabolism; GLU, glucose metabolism; H, hour; HF, heart failure; I/R, ischemia-reperfusion; KB, ketone body metabolism; LAD, left anterior descending coronary artery; Min, minute; Mito, mitochondria structure and functions; NR, not reported; OS, oxidative stress; SGLT2i, sodium-glucose transporter 2 inhibitor; STZ, streptozocin; T2DM: type 2 diabetes mellitus.

**Table 9:** Effects of SGLT2i on cardiac energy metabolism in HF.

Author, year	Disease types/model (methods)	SGLT2i name and dose	Starting time/intervention time of SGLT2i	FA	GLU	KB	Mito	OS
Hunde-rtmark, 2023 (20)	HFrEF and HFpEF	Empa (10 mg/day)	NR/12 weeks	NR	NR	Plasma→	NR	NR
Berezin, 2023 (21)	T2DM and HF	Dapa (10 mg/day)	NR/6 months	NR	NR	NR	NR	NR
Zannad, 2022 (22)	HFrEF and HFpEF	Empa (10 mg/day)	NR/52 weeks	NR	NR	NR	NR	NR
Song, 2021 (31)	Adverse cardiac remodeling (LAD ligation), *Parkin* gene knockout	Empa (10 mg/kg/day)	2 h/2 weeks	NR	NR	NR	Better	NR
Shiraki, 2022 (32)	DCM (Heart and skeletal muscle-specific MnSOD-deficiency)	Empa (10 mg/kg/day)	NR/7 weeks	Heart→	Glucose uptake→, glycolysis↓	Plasma→	Better	NR
Li, 2022 (34)	HF (TAC)	Empa (10 mg/kg/day)	2 weeks/4 weeks	NR	NR	NR	Better	↓
He, 2022 (12)	HFpEF (high-salt diet)	Cana (20 mg/kg/day)	12 weeks/12 weeks	Plasma→, heart↑	Glucose oxidation↓	Plasma↑, Heart↑	Better	↓
Young, 2021 (38)	Cardiac pressure overload (HFD and TAC)	Sota (10 mg/kg/day)	HFD (1 weeks) +TAC (4 weeks)/7 weeks	NR	NR	Plasma→, Heart→	NR	NR
Li, 2021 (41)	Cardiac pressure overload (TAC)	Empa (10 mg/kg/day)	2 weeks/4 weeks	Heart↑	Glucose uptake↓, glucose oxidation↑, glycolysis↓	Plasma→	NR	NR
Li, 2020 (43)	Cardiac pressure overload (AC)	Dapa (10 mg/kg/day)	12 weeks/12 weeks	NR	NR	NR	NR	↓
Yurista, 2019 (44)	MI (LAD ligation)	Empa (30 mg/kg/day)	NR/ Early: 2 days Late: 2 weeks	Heart↑	Glucose oxidation↑	Plasma↑, Heart↑	Better	↓
Santos-Gallego, 2019 (15)	HF (balloon occlusion of LAD)	Empa (10 mg/kg/day)	2 h/8 weeks	Heart↑	Glucose uptake↓, glucose oxidation↓, glycolysis↓	Plasma↑, Heart↑	NR	NR

AC, aortic coarctation; Cana, canagliflozin; Dapa, dapagliflozin; DCM, diabetic cardiomyopathy; Empa, empagliflozin; FA, fatty acid metabolism; GLU, glucose metabolism; H, hour; HF, heart failure; HFD, high-fat diet; HFpEF: heart failure with preserved ejection fraction; HFrEF, heart failure with reduced ejection fraction; KB, ketone body metabolism; LAD, left anterior descending coronary artery; MI, myocardial infarction; Mito, mitochondria structure and functions; MnSOD, manganese superoxide dismutase; NR, not reported; OS, oxidative stress; SGLT2i, sodium-glucose transporter 2 inhibitor; Sota, Sotagliflozin; TAC, transverse aortic constriction; T2DM: type 2 diabetes mellitus.

## 4. Discussion

Many clinical diseases lead to cardiac involvement and cause cardiac energy metabolism disorders. The effects of various diseases on myocardial energy metabolism might vary due to different pathogenesis. SGLT2i has been widely used to treat heart diseases in clinical practice. Although existing studies had shown its cardiovascular benefits, the specific pharmacological effects of SGLT2i on cardiac energy metabolism were not yet clarified. After analyzing 10 clinical and 24 animal studies, we found that SGLT2i optimized the cardiac energy production environment, improved cardiac energy metabolism, and increased cardiac energy efficiency through the pharmacological effects on cardiac FA, glucose, ketone metabolism, mitochondria structure and functions, and oxidative stress. Moreover, we also explored the effects of SGLT2i on cardiac energy metabolism in DM and HF from a disease perspective.

### 4.1. Effects of SGLT2i on cardiac FA metabolism

Under physiological conditions, the heart utilizes FAs (40%–60%) and glucose (20%–40%) for ATP production, while LA, KBs, and amino acids contribute minimally, reflecting the diversity of cardiac metabolic substrates (48–51). FAs are taken up from the circulation into the cardiomyocytes cytoplasm by CD36 and transported into the mitochondria for β-oxidation via CPT-1, from where they enter into the tricarboxylic acid cycle and undergo aerobic oxidation through a series of metabolic enzymes to produce ATP. PGC1-α, PPARα, AMPK and ACC are proteins related to FA metabolism. The myocardial FA oxidation (FAO) rate changes with the progression of diseases. Cardiac FA metabolism decreased in various diseases. Many studies had showed that FAO was downregulated in patients with idiopathic dilated cardiomyopathy (IDCM), Dahl salt-sensitive rats fed a high-salt diet, Wister rats post-MI, and canine model of cardiac pacing (52–59). The decrease in FAO was not a consistent finding. According to several researches, individuals with congestive HF had higher FA uptake, while patients with IDCM showed no differences in FA uptake (60–62). Other studies showed that HFpEF, especially when HFpEF was associated with DM and obesity, upregulated FAO (63–65). T2DM men with cardiomyopathy and obese women with left ventricular hypertrophy exhibited increased myocardial FA uptake and oxidation (49, 64, 65). Moreover, FAO was also increased in murine HFpEF model involving HFD and aging (66). In addition to diseases types, different stages of diseases also caused changes in cardiac FA metabolism. Interestingly, FA metabolism showed temporal dynamic changes in ischemic heart disease-induced HF. In the acute ischemic stage, cardiac FAO was basically normal (67), and FA metabolism gradually decreased with HF progression from the early stage to the decompensated stage (52, 59, 67, 68). While there was a notable rise in cardiac FA uptake and oxidation, during the early stage of DM (65, 69–74). However, with the progression of DM, high rates of cardiac FA β-oxidation negatively affect cardiac efficiency (myocardial oxygen consumption/cardiac work) in humans (74, 75) and animals (76–78). Additionally, the increased FA dependence of the diabetic heart impaired the antioxidant capacity of the mitochondria, cellular ATP shuttle (79, 80), and caused cardiac lipotoxicity initiated by a mismatch between FA uptake and β-oxidation (66, 81–86). Therefore, cardiac FA metabolism might decrease in the end-stage of diabetic cardiomyopathy (DCM) due to the above adverse consequences, leading to a deficiency in cardiac energy metabolism. In conclusion, patterns of FA utilization might be connected with the types and stages of diseases. SGLT2i was closely related to FA metabolism and Empagliflozin had been identified as a regulator of adiposity and energy metabolism in adipose tissue. According to certain researches, SGLT2i prevented tubular epithelial cells from undergoing the metabolic transition from FAO to glycolysis, which is mediated by the hypoxia-inducible factor 1-α (HIF-1α) (87). When glucose is substituted for FAs in podocytes as an energy substrate in experimental Alport syndrome (AS), lipotoxicity is mitigated and renal function is enhanced by Empagliflozin (88).

Importantly, SGLT2i was beneficial for cardiac FA metabolism (11, 15, 41, 44, 89, 90). According to the literatures, 6 animal studies showed that SGLT2i promoted cardiac FA metabolism and the disease models included high-salt diet-induced HFpEF (12), TAC-induced cardiac pressure overload (41), LAD ligation-induced MI (44), HF induced by balloon occlusion of LAD (15), DCM established by intraperitoneal injection of STZ (30), and composite models of DM and I/R (42). Cardiac FA metabolism was decreased in the pathological states, while SGLT2i treatment increased the FA metabolism in 5 animal studies (12, 15, 41, 42, 44). Xi et al. demonstrated that SGLT2i promoted FA metabolism despite its upregulation in the diabetic group (30). The proteomics data suggested that the proteins related to the mechanism of lipid metabolism, such as monocarboxylic acid, cellular lipid and lipoprotein metabolism, as well as FA β-oxidation and lipid transport, were increased in the diabetic group, which was consistent with our descriptions. Also, Empagliflozin group exhibited upregulated levels of proteins related to lipid metabolic processes compared to the diabetic group (30). Hence, the role of SGLT2i in promoting cardiac FA metabolism needed to be investigated further. Among the above 6 animal studies on SGLT2i-promoted cardiac FA metabolism, 4 belonged to HFpEF, and the disease models were DCM created by intraperitoneal injection of STZ (30), HFpEF brought on by high-salt diet (12), TAC-induced cardiac pressure overload (41), and composite models of DM and I/R (42). Although the LAD ligation-induced MI model was generally used to establish HFrEF, the EF value of was >50% with this model in Yurista et al.'s study (44). In the study by Santos-Gallego et al. (15), although the EF value reached the level of HFrEF, the results showed that SGLT2i improved cardiac function and cardiac hypoxia. HFpEF is a multisystem syndrome, with metabolic stress/obesity, hypertension, and aging being the most common comorbidities. Some studies confirmed that the heart of HFpEF was in a state where hypoxia was not severe, while energy deprivation was existing, and resting ATP levels in the heart had been reduced to 20%–40% of the normal (53, 91–94). In the 6 animal studies wherein SGLT2i promoted cardiac FA metabolism (12, 15, 30, 41, 42, 44), the hearts were in a state of energy metabolism: ATP was scarce, but hypoxia was not yet serious, thereby, increasing the total amount of ATP production was crucial. When compared to other substrates, FAO generated the most ATP molecules per mole (129 ATP molecules for complete oxidation of one molecule of palmitate). Therefore, when the hypoxia of the heart was not severe, SGLT2i increased the total ATP production of the whole heart by promoting cardiac FA metabolism, hence improving the adverse situation of cardiac energy deficiency.

However, 3 studies showed that myocardial FA metabolism did not increase with SGLT2i treatment. Trang et al. showed that the levels of proteins related to FAs metabolism were lower in the Empagliflozin group compared to the DM group (13), which might be due to the shorter induction time (2 weeks) of the DM model than the general time (4–8 weeks); therefore, we speculated that SGLT2i inhibited FA uptake and metabolism in the early stage of DM. Lauritsen et al. showed that the relative uptake rate of myocardial FFA was reduced, and the absolute uptake rate was unchanged after Empagliflozin treatment (11). This phenomenon could be attributed to the milder severity of the disease in DM patients (diabetes duration only >1 year and metformin as the only antidiabetic pharmacological treatment), as well as the high FFA and low insulin state during SGLT2i treatment, which increased CD36 degradation and thus decreased myocardial FFA uptake capacity (95, 96). Shiraki et al. used the heart and skeletal muscle-specific manganese superoxide dismutase (MnSOD) deficiency dilated cardiomyopathy mouse model and demonstrated that the amount of cardiac FA uptake and the CD36 content in the Empagliflozin group did not alter significantly compared to the model group (32). Hence, we suspected that this disease model did not affect cardiac FA metabolism, but influenced the oxidative stress of the heart.

Numerous studies had shown that SGLT2i reduce body weight, with the initial weight loss being attributed to osmotic diuresis and urinary glucose (97). By lowering calories and improving hypothalamic insulin response, SGLT2i treatment might cause adipocytes in perivascular adipose tissues to become smaller and promote them to spend more energy. Significant weight loss would follow, along with reductions in visceral fat, subcutaneous fat, and overall adiposity (98–100). But SGLT2i treatment-induced weight loss was limited. The rise in FFA levels was observed to peak at week 24 and then to fall from week 24 to week 52, indicating that the improvement of anti-lipolysis effects might have mitigated the weight loss resulting from long-term SGLT2i medication (101). This review found that SGLT2i could cause weight loss through prompting lipid mobilization and lipolysis in DM patients and animals with metabolic diseases caused by high salt diet, high fat diet or *leptin* gene knockout. However, in patients or animals with HF, dilated cardiomyopathy and other heart diseases or DM patients with short duration of medication (2–4 weeks), the body weight remained unchanged, which might be related to the absence of an increase in body fat mass under the disease state. However, in the STZ-induced DM animal models, STZ only destroyed islet cells without causing an increase of body fat mass, resulting in a significant weight loss in the pathological state. Thus, animal body weight remained unchanged or increased after SGLT2i application.

In conclusion, the changes in cardiac FA metabolism might be connected with the types and stages of diseases; however, SGLT2i enhanced cardiac ATP production by promoting FA metabolism under the condition of poor ATP source but relatively sufficient oxygen, thereby improving the efficiency of cardiac energy production. Moreover, SGLT2i also affect body weight in different diseases.

### 4.2. Effects of SGLT2i on cardiac glucose metabolism

First-choice myocardial fuel, FA, is primarily oxidized by the mitochondria to meet the high energy requirements of a healthy heart. Nonetheless, the contribution of glucose consumption for cardiac ATP production is increased by the elevated levels of insulin and plasma glucose during the feeding state. During moderate-intensity exercise, the glucose uptake and oxidation in cardiomyocytes were also found increased (102–104). Because of its metabolic flexibility, the heart can utilize various substrates based on workload and substrate availability. As another important cardiac energy source, glucose has higher oxygen efficiency, indicating that glucose produces more ATP while consuming an equivalent amount of oxygen. Glucose is mainly absorbed into cardiomyocytes from the circulation by GLUT4 in physiological state, but less by GLUT1. Nevertheless, in pathological conditions, such as cardiac hypertrophy and HF, the heart returned to the fetal phenotype characterized by downregulated GLUT4 and upregulated GLUT1 (105). Following intake, glucose is quickly phosphorylated to glucose 6-phosphate and undergoes a series of metabolic processes. In the cytoplasm, glucose is converted into pyruvate by glycolytic pathway. Under aerobic environment, pyruvate is carried into the mitochondria where PDH decarboxylates it to generate acetyl CoA. Acetyl CoA then proceeds through the tricarboxylic acid cycle to produce ATP. PDH is a key regulator promoting glucose oxidation but is inactivated by PDK4 after phosphorylation (106). When oxygen is insufficient or myocardium hypoxia is severe, pyruvate in the cytoplasm could be glycolysed into LA by LDH, and excessive accumulation of LA in cardiomyocytes could lead to acidosis.

No known SGLT2 receptor was found in the heart (107), however, several studies had revealed a direct protective effect of SGLT2i in HF, with several factors influencing this effect. It had been discovered that SGLT2i might directly inhibit GLUT1 (108). In this systematic review, 3 studies indicated that Empagliflozin reduced cardiac glucose uptake, inducing a shift in the utilization of myocardium substrates from glucose toward other sources (11, 15, 41). The analysis of metabolites revealed a decline in cardiac glucose intake, while the contents of GLUT1 and GLUT4 were not detected. Li et al. discovered that GLUT1 and GLUT4 were highly affinified for Empagliflozin in the heart as assessed by molecular docking. These findings indicated that SGLT2i blocked the direct combination of glucose and GLUT1/4 in the heart, such that the cardiac glucose intake decreased (41). However, we also found that SGLT2i improved the gene and protein levels of GLUT4 (13, 44), although glucose uptake and GLUT1 content were not detected. Lauritsen et al. suggested that SGLT2i stimulated the expression of p-AMPKα2 protein, which significantly enhanced the cardiac energy metabolism (109), and increased AMPK activation, which enhanced the transcription and translocation of GLUT4, thus augmenting GLUT4 gene and protein expression in the heart. Moreover, whether the alterations in glucose uptake and glucose transporters were related to the types of diseases and different stages of the model remained to be further explored and confirmed.

3 animal studies showed that SGLT2i treatment enhanced glucose oxidation in T2DM (13), cardiac pressure overload (41) and MI (44). When mitochondria oxidative metabolism was damaged due to hypoxia, oxidative stress and other pathological factors, such as high glycolysis rate and low glucose oxidation rate could lead to increased decoupling of glycolysis and glucose oxidation, as well as changes in ion homeostasis, thus reducing cardiac energy generation efficiency (110). The current results showed that Empagliflozin increased cardiac glucose oxidation and restored the coupling between glycolysis and glucose oxidation (41, 102). Some studies revealed that after aortic constriction in rats, cardiac glucose oxidation increased in initial stage, while in the later stage of HF, glucose uptake and utilization were reduced. Other studies demonstrated that Empagliflozin restored myocardial glucose oxidation to non-diabetic level in diabetic rats, promoted the normalization of cardiac glucose oxidative metabolism and ATP generation, enhanced cardiac function, and optimized cardiac hypertrophy and fibrosis in non-diabetic rats after MI (44, 76). Nevertheless, 2 studies (12, 15) also concluded that the glucose oxidation decreased after the SGLT2i intervention. One study used Dahl salt-sensitive (DSS) rats fed with 8% high-salt diet for 12 weeks (12), and the other study used balloon occlusion of LAD for 2 h to establish HF model. The main reason for decreased glucose oxidation might be the increase in FA and ketone metabolism after the long-term intervention of SGLT2i (12, 15). Whether this phenomenon was related to the disease types and intervention time needed further investigation. 4 studies confirmed that SGLT2i reduced anaerobic or cardiac glycolytic metabolism, and decreased intracellular LA accumulation and acidosis in cardiomyocytes (15, 32, 39, 41). The lower blood LA level produced by anaerobic metabolism in Empagliflozin group suggested that the cardiac mitochondria was capable of supplying adequate energy for the heart to beat, thus reducing the dependence on the compensatory energy supply of anaerobic metabolism (32). 2 clinical studies found that circulating LA levels were not affected (11, 26), which might be due to the circulating LA level of T2DM patients fluctuate slightly little, and drugs were not sensitive to this index.

Taken together, SGLT2i inhibited cardiac glucose uptake but upregulated the expression of GLUT4. It also increased the proportion of glucose oxidation under most circumstances. In addition, SGLT2i inhibited glycolysis and reduced the occurrence of intracellular LA accumulation and acidosis in cardiomyocytes.

### 4.3. Effects of SGLT2i on cardiac ketone metabolism

Typically, ketone metabolism accounts for approximately 10% of the substrates of cardiac energy metabolism (48, 49, 51, 111). However, KBs are recently recognized as crucial energy substrates for the heart (112, 113). When the heart developed energy metabolism disorders due to various diseases, the normal oxidative metabolism of FAs and glucose was disrupted, making the heart depend on KBs as fuels for metabolism (114–116). Under physiological conditions, KBs are mainly produced by FAs in the liver when supply of carbohydrates is restricted, such as ketogenic diets, fasting, and extended exercise. The human body produces three distinct ketones: β-OHB, acetoacetate (AcAc), and acetone. 80% of the circulating ketone concentration is made up of β-OHB, which is the primary KBs oxidized in the heart (117). MCT1 promotes cardiomyocytes uptake β-OHB, and then, β-OHB passes through a series of metabolic enzymes, such as BDH1 and SCOT, to produce acetyl CoA, which then enters the tricarboxylic acid cycle. KBs had a beneficial effect on hemodynamics and metabolism in individuals with various illnesses, such as overweight, T2DM, ischemic HF and cognitive impairment (118–122). A modest increase in cardiac ketone metabolism was salutary for overall cardiac function. Some studies showed that the infusion of β-OHB or ketone ester improved contractile performance in patients with HFrEF and decreased adverse remodeling in animals with HF (120, 123, 124). The cardiac consumption of β-OHB resulted in a considerable improvement in heart function and membrane potential stability, which in turn strengthened the cardiomyocytes' antiarrhythmic potential (125). Nevertheless, it was challenging to keep the high level of circulating ketone with either ketone or ketone ester infusions. This issue could be resolved by SGLT2i. Although originally developed as an anti-hyperglycemic agents for the treatment of DM, SGLT2i had recently been shown to exert significant cardioprotective effects in HF patients, potentially by increasing circulating KBs and affecting cardiac ketone metabolism (6, 126–128).

Our review showed that SGLT2i enhanced cardiac ketone metabolism and levels of circulating ketone with respect to the already increased ketone level and ketone metabolism under the diseases states. 4 clinical (11, 19, 24, 27) and 6 animal studies (12, 15, 24, 39, 44, 45) demonstrated that SGLT2i increased the circulating level of ketone. The result could be attributed to the following: in one sense, SGLT2i stimulated hepatic ketogenesis by reducing insulin levels, thereby increasing lipolysis and circulating FFA levels. But in another, it might be linked to a lower renal clearance of KBs (29, 129, 130). The elevated levels of circulating KBs promoted cardiac ketone utilization (112, 113), which was confirmed by 4 studies (12, 15, 39, 44) in our systematic review. KB produces ATP in a higher oxygen-efficient manner than FA (P/O 2.5 for KB vs. 2.33 for palmitate), and KB oxidation liberates more energy than glucose (244 kcal/mol for KB vs. 224 kcal/mol for glucose) (114, 115). Thus, KBs are energetically efficient fuels for cardiac energy production which are beneficial to increase cardiac ATP production and improve overall cardiac work efficiency. In addition, KBs, as substrates for mitochondria energy metabolism, do not produce LA, and reduced the risk of intracellular LA accumulation and acidosis in cardiomyocytes. However, no significant change was detected in cardiac ketone metabolism after SGLT2i treatment in 2 studies (24, 38). Both these studies used HFD, which might be the major cause for the lack of significant effect of SGLT2i on cardiac ketone metabolism. SGLT2i acts on the renal proximal tubules, but after HFD, fat is deposited on renal proximal tubules. Increased excretion of KIM-1 in urine, sign of damage to the proximal tubule, had been reported in HFD mice (1). Some studies also reported disrupted sodium handling in HFD mice, suggesting impaired renal proximal function (2). Therefore, renal proximal tubular injury caused by HFD resulted in a decline in the diuretic and glycosuria effects of SGLT2i, and cardiac ketone metabolism remains unaltered ultimately. In addition, 2 clinical (20, 26) and 2 animal studies (32, 41) showed that the plasma ketone levels did not alter significantly after SGLT2i treatment, which might be related to low SGLT2i dose and short treatment time.

Therefore, without affecting the activity site of SGLT2i—the renal proximal tubules, SGLT2i could improve cardiac ketone metabolism, thus increasing the efficiency of ATP production with reducing the occurrence of adverse metabolic consequences such as acidosis, which could reverse the cardiac energy metabolism disorder in the pathological state.

### 4.4. Effects of SGLT2i on mitochondria structure and functions

As an energy-processing plant, the heart generates enormous ATP to sustain systolic and diastolic function from two main sources: mitochondria oxidative phosphorylation and glycolysis. In the physiological state, 95% of myocardial ATP requirements are typically met by mitochondria OXPHOS, with glycolysis providing the remaining 5% (131–134). Cardiomyocytes have more mitochondria than other cell types, therefore, structural damage and dysfunction of the mitochondria inevitably result in decreased cardiac energy production (135). Mitochondria undergo continuous cycles of fusion and fission that regulate mitochondrial homeostasis, recycling, and biogenesis to sustain energy production, hence, the structure and functions of mitochondria depend on the balance between mitochondrial fusion and fission (136). The outer membrane protein mitofusin (MFN) and the inner membrane protein OPA1 are associated with mitochondrial fusion (49, 137, 138). DRP1 and FIS1 are involved in mitochondrial division. In our systematic review, 3 studies (35, 42, 47) confirmed that SGLT2i regulated the fusion and fission of the mitochondria. The disease models used in these 3 studies include acute kidney injury (AKI) induced by bilateral renal artery ischemia (35), I/R in GK rats (42), and MetS induced by high-carbohydrate diet (47). The results demonstrated that the pro-fusion proteins MFN1/2 and OPA1 were upregulated, and the pro-fission proteins DRP1 and FIS1 were downregulated after SGLT2i treatment (35, 42, 47). According to Koizumi et al., Empagliflozin brought the altered atrial protein expression associated with fission and fusion of mitochondria back to levels comparable to those observed in the control group (139). Additionally, 6 studies showed that SGLT2i increased the mitochondria number (34), length (35), pleomorphism (30, 35), mean area (34, 39) and MMP (42, 47) while alleviating mitochondria irregular swelling, fractured cristae and fuzziness (35), implying an improvement in mitochondria structure. Consistent with our results, Mizuno et al. similarly showed that Empagliflozin normalized mitochondria size and quantity in DM hearts following MI (140).

Biogenesis and OXPHOS are two important functions of cardiac mitochondria. Mitochondrial biogenesis is characterized by generating new mitochondria and increasing mitochondrial content and number. Alterations in the mitochondria fusion and fission processes as well as mitophagy suppression might lead to a decrease in mitochondrial biogenesis (141). PGC1-α is a vital transcriptional regulator of mitochondrial biogenesis and can activate the transcriptional expression of nuclear respiratory factor-1/2 (NRF-1/2). These are target genes encoding the proteins that mediate mitochondria replication, maintenance, and the generation of electron transport chain (ETC) components (142). Previous studies had demonstrated that AMPK/SIRT-1/PGC-1α signaling pathway was an energy-sensing network that significantly regulated mitochondrial biogenesis, energy metabolism, and oxidative stress (143). In our review, 4 studies elucidated that SGLT2i increased the proteins or mRNA expression of AMPK, SIRT-1, and PGC-1α in cardiomyocytes, exerting a cardioprotective role by upregulating mitochondrial biogenesis and enhancing OXPHOS (12, 29, 34, 44). The decline in the PGC1-α level might contribute to mitochondria dysfunction observed in HF (144). Acting as an upstream factor, SIRT1 directly stimulated PGC1-α expression (145, 146). Zarei et al. observed that stimulating the SIRT1/PGC1-α pathway reduced the cardiomyocytes toxicity of D-galactose-induced by improving mitochondrial biogenesis (141). According to Santos-Gallego et al., Empagliflozin therapy enhanced heart function and mitochondria energetics in porcine model of HF, which was achieved by upregulating the expression of AMPK and PGC-1α (15). These studies supported the conclusions of our review. SGLT2i was also advantageous for OXPHOS of cardiac mitochondria. In mitochondria, energy is generated via OXPHOS, wherein the electrons are transferred from negative to positive redox potentials along the ETC (147) which consists of 4 proteins: mitochondria respiratory complexes I–IV (COX I–IV), localized on the inner mitochondria membrane. In our systematic review, 3 studies showed that SGLT2i increased the OXPHOS capacity of cardiac mitochondria COX I, II and III (31–33). Upregulated OXPHOS capacity made mitochondria less reliant on anaerobic metabolism's compensatory energy source, allowing cardiac mitochondria to obtain sufficient energy from aerobic metabolism for a continuously beating heart. According to Nambu et al., Empagliflozin ameliorated the reduced skeletal muscle capacity for exercise by restoring the mitochondrial OXPHOS in murine HF (148).

In summary, SGLT2i protected mitochondria structure by maintaining the balance between mitochondrial fusion and fission and restoring mitochondria number, mean area, and MMP. Moreover, SGLT2i protected mitochondria functions by increasing its biogenesis and the ability of OXPHOS, thereby exerting beneficial effects on cardiac energy metabolism.

### 4.5. Effects of SGLT2i on oxidative stress

Sustained oxidative stress and related inflammation play a crucial role in myocardial injury, forming the core pathological link of HF and other cardiovascular diseases (149, 150). The re-oxidation of myocardial tissue led to excessive ROS and increased oxidative stress, which induced the break of metabolic homeostasis. It has long been recognized that the primary source of ROS generated by NOX is the mitochondria. Among the members of the NOX family that transport electrons across the plasma membrane and produce superoxide (151), cardiomyocytes primarily express NOX2 and NOX4 (152, 153). Under physiological conditions, ROS advocates the basic signaling function of regulating mitochondria activity and cell adaptation to stressors. However, when cardiac mitochondria were damaged, ROS production was enhanced. Long-term exposure to oxidative stress and ROS accumulation had been linked to reduced antioxidant capacity, imbalanced redox signals, and macromolecular damage, finally, ROS-mediated myocardial injury and cardiomyocytes death was effectuated (154, 155). Also, excessive ROS led to defective myocardial remodeling and impaired myocardial contractility, that in turn aggravated HF (156). Some studies showed that hyperglycemia stimulated ROS production, and the excessive ROS might lead to the long-term development of diabetic complications (157, 158).

The oxidative stress produced by the diseases reported in 11 studies was shown to be mitigated in cardiomyocytes by the application of SGLT2i. By activating the AMPK/SIRT-1/PGC-1α pathway, Empagliflozin protected mitochondria functions and reduced oxidative stress (29). The enhanced protein expression of SIRT1 inhibited ROS accumulation, NOX activity and MDA content, and upregulated the activities of antioxidant enzymes like SOD and SDH, increasing endogenous heart antioxidant capacity and alleviating oxidative stress (29, 36, 43). In addition, the expression of AOPP and NOX2 was suppressed by Empagliflozin in the rat model of non-diabetic MI (44). Canagliflozin directly inhibited oxidative stress through SGLT1/AMPK/Rac1 signaling and played a crucial role in suppressing the pro-inflammatory and pro-apoptotic effects of TNF-α, IL-1, and NF-κb pathways (23). Shen et al. showed that Dapagliflozin reduced oxidative stress and regulated the survival, apoptosis, growth of cardiomyocytes through PI3K/Akt signaling pathway (37). Also, chronic administration of Empagliflozin induced dimerization of p(Y705) STAT-3, upregulated the downstream proteins SOD2 and VEGF, and reduced the cardiac oxidative stress response after I/R injury in non-diabetic mice (40). Altogether, SGLT2i played an antioxidant role by reducing cardiac oxidative stress for a direct cardioprotective effect.

### 4.6. Effects of SGLT2i on cardiac energy metabolism in DM and HF

In DM patients, myocardial metabolism flexibility was impaired, FA β-oxidation became the main source of ATP generation, and glucose oxidation was significantly reduced. These changes had been reported in preclinical models, T1DM and T2DM patients (65, 70, 72–74, 159). SGLT2i promotes urine glucose excretion, which is not affected by islet function and insulin resistance (160). Several clinical studies illustrated that patients with T2DM and atherosclerotic diseases reflected a low HF hospitalization rate and mortality under SGLT2i treatment (161–163). In the 34 included studies, 11 studies were associated with DM (7 clinical and 4 animal studies). The diseases in the clinical studies involved T2DM, pre-diabetic insulin resistance, and T2DM complicated with HF, and the disease models in animal studies included DM, DCM and insulin resistance. These clinical and animal studies related to DM showed that the cardiac glucose intake remained unchanged or decreased but the cardiac glucose oxidation and the KBs levels in circulation were elevated with treatment of SGLT2i. The reason might be attributed to the fact that SGLT2i reduced the dependence on FA metabolism and avoided lipotoxicity-mediated myocardial damage by improving the oxidative utilization of glucose (13, 164). Some studies demonstrated that SGLT2i reduced the blood glucose concentration through reduced reabsorption of glucose by the kidney, thus treating T2DM (45, 165). However, we found the FA metabolism in the myocardium was inhibited after SGLT2i treatment for 4 weeks in DM, but was upregulated after treatment for 12 weeks in DCM and after a single intravenous administration in a composite model of DM and I/R, which might be related to models types, stages of DM development, and the intervention time of SGLT2i. Thereby, we discovered that the application of SGLT2i generally promoted the conversion of cardiac metabolism substrates, increased cardiac glucose oxidation and ketone metabolism, regulated FA metabolism to the direction of benefit to the whole body according to the disease states, and finally restored the balance between myocardial energy demand and supply.

The alteration of cardiac energy metabolism is one of the core pathological mechanisms of HF. The energy metabolism alterations in HF are complex and depend on the severity and types of HF as well as the coexistence of common comorbidities, such as obesity and T2DM. An energy deficit is present in the failing heart, and the relative contribution of various metabolism substrates is also changed compared to the physiological state. Of the 34 included studies, 12 studies were associated with HF (3 clinical and 9 animal studies). The analysis showed that SGLT2i increased cardiac FA (12, 15, 41, 44) and ketone metabolism (12, 15, 44), with increasing (41, 44) or decreasing (12, 15) glucose oxidation and decreasing glycolysis (15, 32, 41). 2 studies showed that SGLT2i reduced the rate of cardiac glucose oxidation (12, 15), which could be due to increased metabolism of FAs and KBs produced more abundant ATP. Typically, cardiac glucose uptake and glycolysis were increased in HF, which was linked to the elevated GLUT1 protein expression (166, 167). Some studies showed that cardiac glucose oxidation and FAO in HF influenced each other, and the changes were uncertain, which were dependent on the types of HF. For example, myocardial glucose oxidation was reduced and FAO was increased in HF associated with DM and obesity (63, 64, 168–170); in some researches using mouse models of HF with lower FAO rates, the proportional contribution of glucose oxidation to tricarboxylic acid cycle flux might be increased (171). Recent studies demonstrated that myocardial KB oxidation was increased in HF (172–174), which might be related to the inhibition of FAO, reflecting the mutual regulation of increased KB oxidation and FAO. These metabolic changes ultimately reduced the heart's efficiency of producing energy, while SGLT2i exerted a benign regulatory effect on cardiac energy metabolism in HF. In a word, the results indicated that SGLT2i increased cardiac utilization and metabolism of FAs and KBs in HF, regulated glucose oxidation, and increased cardiac ATP production, exerting beneficial effects on cardiac energy metabolism.

In clinical researches, patients had a disadvantage due to the diversity of their pathological conditions and confounding factors compared to the advantages of controllable research conditions of experimental animals in basic researches. However, clinical patients could reflect the real situations of the diseases. Therefore, it was necessary to combine the two for comprehensive analysis. Moreover, the clinical patients’ indicators were limited to blood lipids, blood glucose, blood ketone, and blood LA, and lack heart-specific metabolic markers, so the changes of indicators were not sensitive enough. Animal studies could detect the levels of proteins closely associated to the cardiac metabolism of FA, glucose, and KB, which directly and pertinently represented the alterations in cardiac metabolism of energy. Overall, SGLT2i's effects on cardiac metabolic indicators in clinical patients and experimental animals were basically consistent, however, the circulating LA level of clinical patients was generally unchanged, while that in the animal heart was significantly decreased.

It was uncertain whether the role of SGLT2i differed in gender. Previous studies had shown that SGLT2i reduced adverse cardiovascular events in both sexes (175), but there had been some reports showed that sex differences might contribute to different responsiveness to SGLT2i (176, 177). In 10 clinical studies of this review, the ratio of male to female was balanced. However, male animals were used in all but one of the literature which used female pigs as research subjects. Moreover, the effect of SGLT2i on gender was not addressed in 34 studies we included, thus more researches is still needed.

### 4.7. SGLT2i adverse effects

SGLT2 inhibitors are generally well-tolerated, but like any medication, they can have adverse effects. Common side effects include increased urination (due to their diuretic effect), urinary tract infections, genital mycotic infections, and a slightly increased risk of hypotension and dehydration, especially in certain populations. Of the 10 clinical studies in this systematic review, only 1 study reported adverse effects after taking SGLT2i, which were not different from those in the placebo group. It showed that the treatment with SGLT2i was well tolerated and remains positive of the overall risk-benefit profile.

## 5. Limitations

Nevertheless, the present study had some limitations. Firstly, the disease models included in the literatures were diverse and not limited to cardiovascular diseases, resulting in the heart might not be the main lesion site and the energy metabolism of the heart might not be affected. Secondly, the main research purpose of some studies was whole-body energy metabolism; thus, not every study contained indicators closely related to cardiac energy metabolism. Finally, we were unable to draw accurate conclusions from the meta-analysis because we employed qualitative synthesis rather than quantitative methods due to the diversity of models and the inconsistency of interventions.

## 6. Conclusions

Among the 34 included studies (including 10 clinical and 24 animal studies), the effects of SGLT2i on cardiac energy metabolism were described in different diseases, such as HF, T2DM, I/R injury, MI, cardiac pressure overload, dilated cardiomyopathy, SCM, DCM, MetS, CA, and other diseases. The findings of the current systematic review demonstrated that SGLT2i reduced cardiac glucose uptake and glycolysis, increased FA metabolism of the heart in most disease states, upregulated ketone metabolism, improved the structure and functions of myocardial mitochondria, and alleviated oxidative stress of cardiomyocytes in all the studies. However, the effects of SGLT2i on FA and glucose metabolism were not consistent due to the different diseases types, stages, and the time of SGLT2i intervention. SGLT2i increased cardiac glucose oxidation in DM and FA metabolism in HF. In addition, it could adjust cardiac FA metabolism in DM and cardiac glucose oxidation of in HF according to the different diseases stages to optimize the heart’ metabolic condition. All of the SGLT2i effects listed above on the regulation of energy metabolism could increase cardiac ATP production and improving cardiac work efficiency. This systematic review discussed the specific effects of SGLT2i on cardiac energy metabolism in detail, providing clinical values for the application of SGLT2i against diseases related to cardiac energy metabolism disorders.

## Data Availability

The original contributions presented in the study are included in the article/**Supplementary Material**, further inquiries can be directed to the corresponding author.
