# Supplementary material for: The changes of cardiac energy metabolism with sodium-glucose transporter 2 inhibitor therapy
Source: Front Cardiovasc Med. 2023 Dec 6;10:1291450. doi: 10.3389/fcvm.2023.1291450 (PMC10731052; doi:10.3389/fcvm.2023.1291450)
Supplement: Supplementary file 1 [file Table1.docx]

Supplementary Material

# Supplementary Data

# Search Strategy：((("Sodium-Glucose Transporter 2 Inhibitors"[Mesh]) OR (((((((((((((Sodium Glucose Transporter 2 Inhibitors[Title/Abstract]) OR (SGLT-2 Inhibitors[Title/Abstract])) OR (SGLT 2 Inhibitors[Title/Abstract])) OR (SGLT2 Inhibitors[Title/Abstract])) OR (Sodium-Glucose Transporter 2 Inhibitor[Title/Abstract])) OR (Sodium Glucose Transporter 2 Inhibitor[Title/Abstract])) OR (SGLT2 Inhibitor[Title/Abstract])) OR (Inhibitor, SGLT2[Title/Abstract])) OR (Gliflozins[Title/Abstract])) OR (Gliflozin[Title/Abstract])) OR (SGLT-2 Inhibitor[Title/Abstract])) OR (Inhibitor, SGLT-2[Title/Abstract])) OR (SGLT 2 Inhibitor[Title/Abstract])) OR (Empagliflozin[Title/Abstract])) OR (Dapagliflozin[Title/Abstract])) OR (Canagliflozin[Title/Abstract])) OR (Ertugliflozin[Title/Abstract]))) AND ((("Myocardium"[Mesh]) OR (((((((((Cardiac Muscle[Title/Abstract]) OR (Muscle, Heart[Title/Abstract])) OR (Heart Muscle[Title/Abstract])) OR (Heart Muscles[Title/Abstract])) OR (Muscles, Heart[Title/Abstract])) OR (Myocardia[Title/Abstract])) OR (Muscle, Cardiac[Title/Abstract])) OR (Cardiac Muscles[Title/Abstract])) OR (Muscles, Cardiac[Title/Abstract]))) OR ((hearts[Title/Abstract]) OR ("Heart"[Mesh])))) AND (("Energy Metabolism"[Mesh]) OR ((((((((((Energy Metabolism[Title/Abstract]) OR (Energy Metabolisms[Title/Abstract])) OR (Metabolism, Energy[Title/Abstract])) OR (Metabolisms, Energy[Title/Abstract])) OR (Energy Expenditure[Title/Abstract])) OR (Energy Expenditures[Title/Abstract])) OR (Expenditure, Energy[Title/Abstract])) OR (Expenditures, Energy[Title/Abstract])) OR (Bioenergetics[Title/Abstract])) OR (Bioenergetic[Title/Abstract])))

# Supplementary Tables

| **Author,**  **Year** | **Study design** | **Sample size** | **Selection bias** | | **Performance bias** | | | **Detection bias** | **Attrition bias** | **Reporting bias** | **Other bias** |
| --- | --- | --- | --- | --- | --- | --- | --- | --- | --- | --- | --- |
|  |  |  | random sequence generation | allocation concealment | | blinding of participants and personnel | | Blinding of outcome assessment | Incomplete outcome data | Selective reporting | Other sources of bias |
| Veelen,2023 [20] | RCT | 14 | low risk | unclear risk | | | low risk | unclear risk | low risk | low risk | low risk |
| Hundertmark,2023 [21] | RCT | 72 | low risk | low risk | | | low risk | unclear risk | low risk | low risk | low risk |
| Gaborit,2021 [25] | RCT | 54 | low risk | low risk | | | unclear risk | unclear risk | low risk | low risk | low risk |
| Lauritsen,2021 [11] | RCT | 13 | low risk | unclear risk | | | low risk | unclear risk | low risk | low risk | low risk |
| Oldgren,2020 [27] | RCT | 53 | low risk | unclear risk | | | low risk | unclear risk | low risk | low risk | low risk |

**Table S1** Qualitative evaluation of RCT studies according to Cochrane ROB tool

**Table S2 *Qualitative evaluation of cohort studies according to NOS criteria***

| **Author,**  **Year** | **QI** | **Q2** | **Q3** | **Q4** | **Q5** | **Q6** | **Q7** | **Q8** | **Total score** |
| --- | --- | --- | --- | --- | --- | --- | --- | --- | --- |
| Berezin,2023 [22] | 1 | 1 | 0 | 1 | 2 | 0 | 1 | 0 | 6 |
| Thirunavukarasu,2021 [26] | 1 | 1 | 0 | 1 | 2 | 1 | 1 | 1 | 8 |
| Polidori,2017 [28] | 1 | 1 | 0 | 1 | 2 | 0 | 1 | 1 | 7 |

**Table S3 Qualitative evaluation of cross-sectional studies according to NIH Risk bias tool**

| **Author,**  **Year** | **QI** | **Q2** | **Q3** | **Q4** | **Q5** | **Q6** | **Q7** | **Q8** | **Q9** | **Q10** | **Q11** | **Q12** | **Q13** | **Q14** | **Total score** |
| --- | --- | --- | --- | --- | --- | --- | --- | --- | --- | --- | --- | --- | --- | --- | --- |
| Zannad,2022 [23] | 1 | 1 | 0 | 1 | 0 | 1 | 1 | 0 | 1 | 1 | 1 | 0 | 0 | 0 | 8 |
| Kondo,2021 [24] | 1 | 1 | 1 | 1 | 0 | 1 | 0 | 1 | 1 | 0 | 1 | 0 | 1 | 0 | 9 |

**Table S4 Qualitative evaluation of animal studies according to SYRCLE’s ROB tool**

| **Author, Year** | **Selection Bias** | | | | **Performance bias** | | **Detection bias** | | **Attrition bias** | **Reporting bias** | **Other** |
| --- | --- | --- | --- | --- | --- | --- | --- | --- | --- | --- | --- |
|  | | **Sequence generation** | **Baseline characteristics** | **Allocation concealment** | **Random housing** | **Blinding** | **Random outcome assessment** | **Blinding** | **Incomplete outcome data** | **Selective outcome reporting** | **Other sources of bias** |
| Croteau, 2023 [29] | | Unclear Risk | Unclear Risk | Unclear Risk | Unclear Risk | Unclear Risk | Unclear Risk | Unclear Risk | High Risk | Low Risk | Low Risk |
| Chen, 2023 [30] | | Unclear Risk | Unclear Risk | Unclear Risk | Low Risk | Unclear Risk | Unclear Risk | Unclear Risk | High Risk | Low Risk | Low Risk |
| Xi, 2022 [31] | | Unclear Risk | Unclear Risk | Unclear Risk | Unclear Risk | Unclear Risk | Unclear Risk | Unclear Risk | High Risk | Low Risk | Low Risk |
| Song, 2021 [32] | | Unclear Risk | Unclear Risk | Unclear Risk | Unclear Risk | Unclear Risk | Unclear Risk | Unclear Risk | Unclear Risk | Low Risk | Low Risk |
| Shiraki, 2022 [33] | | Unclear Risk | Unclear Risk | Unclear Risk | Low Risk | Unclear Risk | Unclear Risk | Unclear Risk | High Risk | Low Risk | Low Risk |
| Nikolaou, 2022 [34] | | High Risk | Unclear Risk | Unclear Risk | Unclear Risk | Unclear Risk | Unclear Risk | Unclear Risk | Unclear Risk | Low Risk | Low Risk |
| Li, 2022 [35] | | Unclear Risk | Unclear Risk | Unclear Risk | Unclear Risk | Unclear Risk | Unclear Risk | Unclear Risk | Unclear Risk | Low Risk | Low Risk |
| He, 2022 [12] | | Unclear Risk | Unclear Risk | Unclear Risk | Low Risk | Unclear Risk | Unclear Risk | Unclear Risk | High Risk | Low Risk | Low Risk |
| Cai, 2022 [36] | | Unclear Risk | Unclear Risk | Unclear Risk | Low Risk | Unclear Risk | Unclear Risk | Unclear Risk | Unclear Risk | Low Risk | Low Risk |
| Zhang, 2022 [37] | | Unclear Risk | Unclear Risk | Unclear Risk | Unclear Risk | Unclear Risk | Unclear Risk | Unclear Risk | Unclear Risk | Low Risk | Low Risk |
| Shen, 2022 [38] | | Unclear Risk | Unclear Risk | Unclear Risk | Low Risk | Unclear Risk | Unclear Risk | Unclear Risk | Unclear Risk | Low Risk | Low Risk |
| Young, 2021 [39] | | Unclear Risk | Unclear Risk | Unclear Risk | Low Risk | Unclear Risk | Unclear Risk | Unclear Risk | Unclear Risk | Low Risk | Low Risk |
| Trang, 2021 [13] | | Unclear Risk | Unclear Risk | Unclear Risk | Unclear Risk | Unclear Risk | Unclear Risk | Unclear Risk | Unclear Risk | Low Risk | Low Risk |
| Tan, 2021 [40] | | Unclear Risk | Unclear Risk | Unclear Risk | Unclear Risk | Unclear Risk | Unclear Risk | Unclear Risk | High Risk | Low Risk | Low Risk |
| Nikolaou, 2021 [41] | | High Risk | Unclear Risk | Unclear Risk | Unclear Risk | Unclear Risk | Unclear Risk | Unclear Risk | High Risk | Low Risk | Low Risk |
| Li, 2021 [42] | | High Risk | Unclear Risk | Unclear Risk | Low Risk | Unclear Risk | Unclear Risk | Unclear Risk | High Risk | Low Risk | Low Risk |
| Gaborit, 2021 [25] | | Unclear Risk | Unclear Risk | Unclear Risk | Low Risk | Unclear Risk | Unclear Risk | Unclear Risk | High Risk | Low Risk | Low Risk |
| Bai, 2021 [43] | | Unclear Risk | Unclear Risk | Unclear Risk | Low Risk | Unclear Risk | Unclear Risk | Unclear Risk | High Risk | Low Risk | Low Risk |
| Li, 2020 [44] | | Unclear Risk | Unclear Risk | Unclear Risk | Unclear Risk | Unclear Risk | Unclear Risk | Unclear Risk | Low Risk | Low Risk | Low Risk |
| Yurista, 2019 [45] | | Unclear Risk | Unclear Risk | Unclear Risk | Low Risk | Unclear Risk | Unclear Risk | Unclear Risk | Unclear Risk | Low Risk | Low Risk |
| Santos-Gallego, 2019 [15] | | Unclear Risk | Unclear Risk | Unclear Risk | Unclear Risk | Unclear Risk | Unclear Risk | Unclear Risk | High Risk | Low Risk | Low Risk |
| Adingupu, 2019 [46] | | Low Risk | Unclear Risk | Unclear Risk | Unclear Risk | Unclear Risk | Unclear Risk | Unclear Risk | Low Risk | Low Risk | Low Risk |
| Uthman, 2018 [19] | | Unclear Risk | Unclear Risk | Unclear Risk | Low Risk | Unclear Risk | Unclear Risk | Unclear Risk | Unclear Risk | Low Risk | Low Risk |
| Durak, 2018 [47] | | Unclear Risk | Unclear Risk | Unclear Risk | Unclear Risk | Unclear Risk | Unclear Risk | Unclear Risk | Unclear Risk | High Risk | Low Risk |
